# Inhibition of nonsense-mediated decay rescues p53β/γ isoform expression and activates the p53 pathway in MDM2-overexpressing and select p53-mutant cancers

**DOI:** 10.1016/j.jbc.2021.101163

**Published:** 2021-09-03

**Authors:** Jayanthi P. Gudikote, Tina Cascone, Alissa Poteete, Piyada Sitthideatphaiboon, Qiuyu Wu, Naoto Morikawa, Fahao Zhang, Shaohua Peng, Pan Tong, Lerong Li, Li Shen, Monique Nilsson, Phillip Jones, Erik P. Sulman, Jing Wang, Jean-Christophe Bourdon, Faye M. Johnson, John V. Heymach

**Affiliations:** 1 Department of Thoracic Head and Neck Medical Oncology, The University of Texas MD Anderson Cancer Center, Houston, Texas, USA; 2 Department of Melanoma Medical Oncology, The University of Texas MD Anderson Cancer Center, Houston, Texas, USA; 3 Department of Bioinformatics and Computational Biology, The University of Texas MD Anderson Cancer Center, Houston, Texas, USA; 4 Institute for Applied Cancer Science, The University of Texas MD Anderson Cancer Center, Houston, Texas, USA; 5 Department of Radiation Oncology and Brain and Spine Tumor Center, Laura and Isaac Perlmutter Cancer Center, NYU Langone School of Medicine, New York, New York, USA; 6 The University of Texas MD Anderson Cancer Center Graduate School of Biomedical Sciences, Houston, Texas, USA; 7 Cellular Division, Ninewells Hospital Campus, School of Medicine, University of Dundee, Dundee, UK

**Keywords:** p53, mRNA decay, alternative splicing, cancer therapy, RNA degradation, targeting NMD, p53β/γ restoration, MDM2, DMSO, dimethyl sulfoxide, GBM, glioblastoma multiforme, IR, ionizing radiation, NMD, nonsense-mediated decay, NMDi, NMD inhibitor, NSCLC, non–small cell lung cancer, PTC, premature termination codon

## Abstract

Inactivation of p53 is present in almost every tumor, and hence, p53-reactivation strategies are an important aspect of cancer therapy. Common mechanisms for p53 loss in cancer include expression of p53-negative regulators such as MDM2, which mediate the degradation of wildtype p53 (p53α), and inactivating mutations in the TP53 gene. Currently, approaches to overcome p53 deficiency in these cancers are limited. Here, using non–small cell lung cancer and glioblastoma multiforme cell line models, we show that two alternatively spliced, functional truncated isoforms of p53 (p53β and p53γ, comprising exons 1 to 9β or 9γ, respectively) and that lack the C-terminal MDM2-binding domain have markedly reduced susceptibility to MDM2-mediated degradation but are highly susceptible to nonsense-mediated decay (NMD), a regulator of aberrant mRNA stability. In cancer cells harboring MDM2 overexpression or TP53 mutations downstream of exon 9, NMD inhibition markedly upregulates p53β and p53γ and restores activation of the p53 pathway. Consistent with p53 pathway activation, NMD inhibition induces tumor suppressive activities such as apoptosis, reduced cell viability, and enhanced tumor radiosensitivity, in a relatively p53-dependent manner. In addition, NMD inhibition also inhibits tumor growth in a MDM2-overexpressing xenograft tumor model. These results identify NMD inhibition as a novel therapeutic strategy for restoration of p53 function in p53-deficient tumors bearing MDM2 overexpression or p53 mutations downstream of exon 9, subgroups that comprise approximately 6% of all cancers.

P53 is a transcription factor, and by binding to specific DNA sequences, it activates transcription of a large number of targets. p53 is considered as a “cellular gate keeper” mediating the cellular response to stressful conditions. Proteins encoded by p53 targets are involved in tumor suppressive pathways including apoptosis, cell cycle arrest, DNA repair, senescence, and metabolism (1, 2). Loss of p53 function is the most common alteration in cancer and occurs *via* multiple mechanisms including *TP53* gene mutations and degradation of WT p53 proteins by its negative regulators MDM2 and HPV-E6 (3, 4). MDM2, the key negative regulator of p53 protein, is a E3 ubiquitin ligase, and by binding to p53, it ubiquitinates p53 for proteasomal degradation (5, 6). Abnormal upregulation of MDM2 due to gene amplification and increased transcription and translation leads to increased p53 protein degradation, causing p53 deficiency in many cancers despite harboring WT p53 (7). Approaches currently under investigation to inhibit MDM2 and reactivate p53 pathway include small molecule inhibitors of MDM2 and gene therapy (8, 9), although none have yet been validated as a therapeutic approach in the clinic. Development of therapeutic approaches for restoration of p53 tumor suppressive function in MDM2-overexpressing cancers is therefore a critical unmet need.

Nonsense mutations in *TP53* leading to p53 inactivation are very common in cancers (10). Nonsense mutations in the coding region generate premature translation termination codons (PTCs), and such transcripts are typically degraded by nonsense-mediated decay (NMD), an RNA surveillance pathway (11). Other than mutations, PTCs can also be generated owing to errors in transcription/splicing, alternative splicing, and gene rearrangements. NMD also regulates the expression of such physiological transcripts (11). Core NMD factors include UPF1, UPF2, and UPF3 proteins. During translation, upon encountering PTC on the mRNA, UPF1 is recruited, which then undergoes a cycle of phosphorylation and dephosphorylation triggering the degradation of the mRNA. Phosphorylation of UPF1 is mediated by SMG-1, a kinase belonging to the phosphoinositide 3-kinase–related kinase family, and its dephosphorylation is mediated by SMG5/SMG7 (11).

*TP53* encodes 12 distinct isoforms (12), among which p53β and p53γ generated by alternative splicing of intron 9 retain key functions of wildtype p53 (12, 13, 14, 15). Unlike full-length p53α, these isoforms lack the C-terminal negative regulatory region containing protein degradation signals (ubiquitinated lysines at positions 370, 372, 373, 381, 382 and 386 (16, 17, 18, 19)) and, hence, are less susceptible to MDM2-mediated degradation (20). However, alternative splicing of p53 intron 9 leads to the generation of PTC in exon 9β and 9γ of p53β and p53γ, respectively, making them susceptible for degradation by NMD. Consistent with this, the p53β isoform was shown to be NMD susceptible (19, 21). Of interest, although p53γ has similar NMD-triggering features as p53β, its NMD susceptibility has not been shown.

In this report, we hypothesized that, owing to the presence of a PTC in exon 9γ, p53γ is also NMD susceptible and that modulating NMD upregulates p53β/γ isoforms and promotes p53 pathway activation overcoming p53 deficiency. Furthermore, this may serve as a potential therapeutic approach to restore p53 in p53-deficient cancers including those in which MDM2 is overexpressed and those with nonsense/frameshift mutations at the C-terminal end of *TP53*. We tested our hypothesis using MDM2-overexpressing non–small cell lung cancer (NSCLC) and glioblastoma multiforme (GBM) cells bearing WT p53 and p53 mutant NSCLC and breast cancer and urinary bladder cancer cells as models.

## Results

### NMD inhibition stabilizes p53β/γ isoforms and activates p53 pathway in MDM2-overexpressing cancer cells

**Figure 1:**
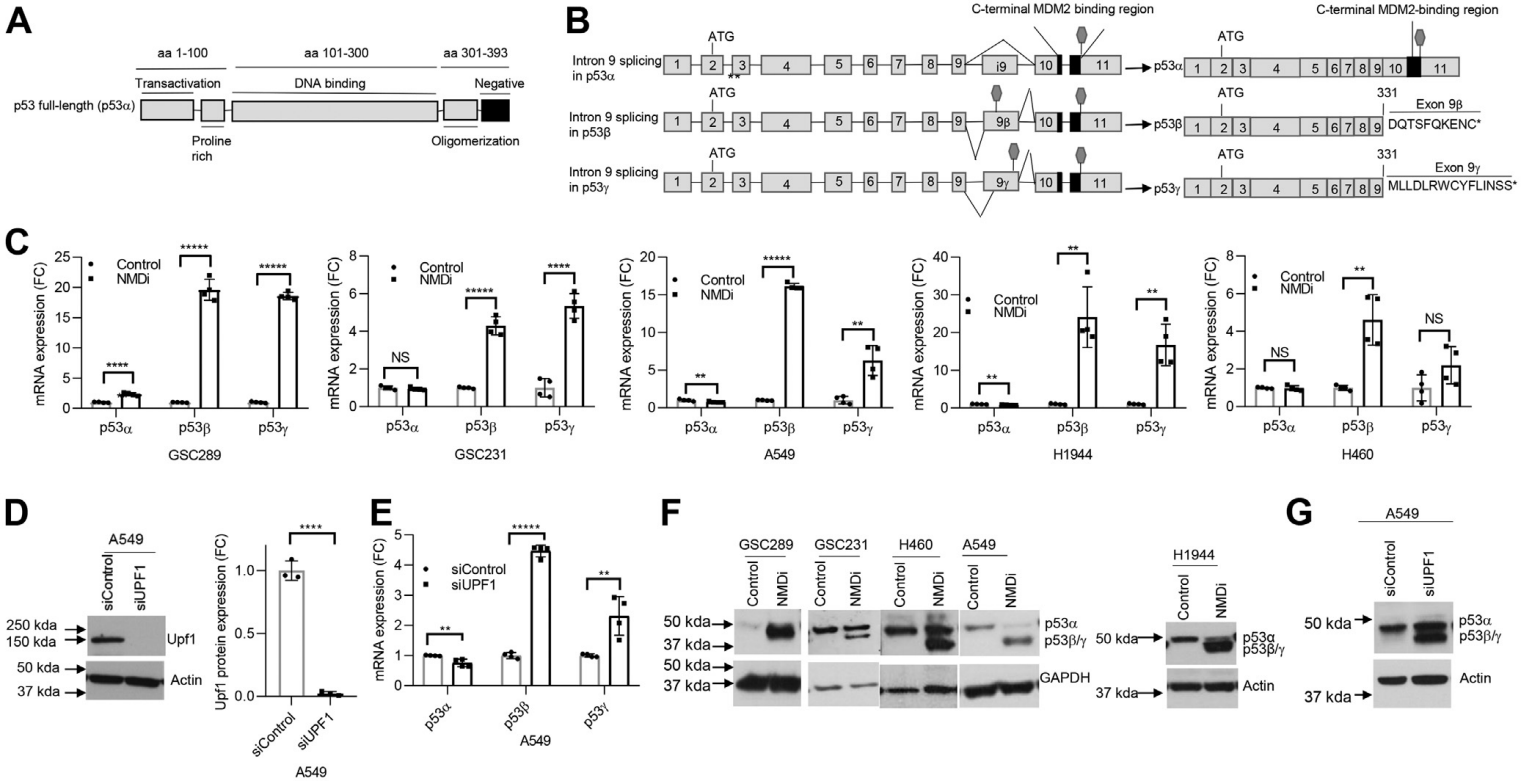
**NMD inhibition induces p53β/γ isoforms in MDM2-overexpressing cancer cells.***A*, schematic showing functional domains (*black lines*) in p53 protein; aa represents amino acids spanning each domain. *B*, schematic of p53α and β/γ isoforms generated by alternative splicing of intron 9. C-terminal MDM2-binding region is depicted in *black*. Stop signs in exons i9 indicate premature termination codons acquired by alternative splicing. Amino acids shown at the C terminus of p53β and p53γ are derived from intron 9. *C*, FC in the mRNA expression of p53α, p53β, and p53γ upon NMDi treatment in the shown cell lines. Cells were treated with either dimethyl sulfoxide (control) or NMDi for 6 h (A549) or 16 h (H1944, H460, GSC231, and GSC289). *D*, Western blot (*left panel*) and its quantification (*right panel*) showing UPF1 knockdown efficiency in A549 cell line treated with either sicontrol or siUPF1. *E*, mRNA expression FC of p53α, p53β, and p53γ in A549 treated with the indicated siRNAs. *F* and *G*, Western blots showing induction of truncated p53 similar in size to p53β/γ proteins in NMDi- or siUPF1-treated cells, respectively (quantifications in Fig. S3, *A* and *B*). RT-qPCR analysis shown (n = 4) are from two independent experiments (two technical repeats from each). Mean ± SD, *p* values, two tailed t-tests, ∗ ≤0.05, ∗∗ <0.01, ∗∗∗ <0.001, ∗∗∗∗ <0.0001, ∗∗∗∗∗ <0.00001, NS, not significant (*p* value > 0.05). FC, fold change; NMD, nonsense-mediated decay; NMDi, NMD inhibitor.

*MDM2* is amplified in about 3.7% of all cancers, including ∼4% of lung cancers and ∼8% of GBM (Table S1). To investigate whether NMD inhibition stabilizes the mRNA expression of p53β/γ isoforms and generates p53β/γ proteins lacking the negative regulatory region (Fig. 1, *A* and *B*), we used NSCLC cell lines (A549, H1944, and H460) and GBM cell lines (GSC289 and GSC231) bearing WT *TP53* and MDM2 overexpression (Fig. S1, *A* and *B*). We inhibited NMD in these cells by treating the cells with the NMD inhibitor IACS14140 (compound 11j (22), henceforth referred to as NMDi), a pyrimidine derivative, which at 1 μM concentration selectively and completely inhibits the phosphorylation of UPF1 by SMG1 (22), a critical step for NMD (11, 22, 23, 24). NMDi treatment and subsequent mRNA expression analysis using isoform-specific primers (Table S2) indicated a significant increase in p53β in all cell lines, whereas p53γ was upregulated significantly in the majority (Fig. 1*C*). NMD inhibition did not increase the mRNA expression of p53α in four of five cell lines tested (Fig. 1*C*). Intriguingly, in GSC289, p53α was also upregulated, although to a much lesser extent compared with p53β and p53γ, upon NMD inhibition (Fig. 1*C*).

To gain additional evidence about the NMD susceptibility of p53β and p53γ, we depleted the key NMD factor UPF1 (11, 23), in A549 and H460 cells and analyzed p53α, p53β, and p53γ mRNA expression. UPF1 depletion significantly increased the mRNA expression of p53β and p53γ in A549 and p53β in H460, but not p53α (Fig. 1, *D* and *E* and Fig. S2, *A* and *B*). mRNA decay analysis in NMDi-treated A549 cells indicated that NMD inhibition prolonged the decay of both p53β and p53γ but not p53α transcript (Fig. S2*C*). These results confirm that p53β and p53γ are NMD susceptible and inhibiting NMD stabilizes these isoforms. Next, we evaluated whether p53β and p53γ mRNAs are translated. Western blot analysis revealed the expression of a truncated p53 protein of approximately 47 to 48 kDa, consistent with the predicted size of p53β/γ, only in the NMDi-treated but not in control cells (Fig. 1*F* and Fig. S3*A*). As in the case of NMDi, Upf1 depletion also induced a truncated p53 protein similar in size to p53β/γ (Fig. 1*G* and Fig. S3, *B* and *C*).

**Figure 2:**
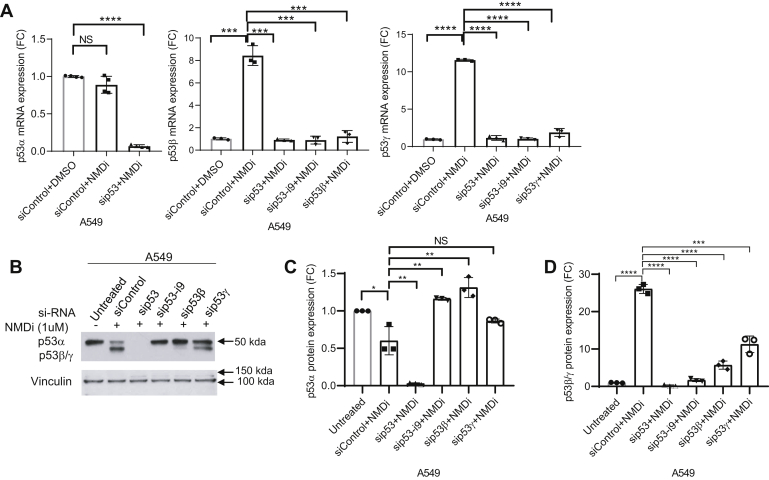
**RNAi-mediated knockdown confirms the identity of p53β/γ.***A*, p53α, p53β, and p53γ mRNA expression FC upon NMD inhibition in A549 cells treated with the indicated siRNAs. Cells treated with siRNAs shown were treated with either dimethyl sulfoxide (control) or NMDi for 16 h. *B*, Western blot analysis showing expression of p53α, p53β, and p53γ isoforms in A549 cells treated with the indicated siRNAs and NMDi. *C* and *D*, p53α and p53β/γ protein quantification in cells treated with the indicated siRNAs. RT-qPCR and Western blot analysis quantifications shown (n = 3) are from three independent experiments. Mean ± SD, *p* values, two-tailed *t* tests, ∗ ≤0.05, ∗∗ <0.01, ∗∗∗ <0.001, ∗∗∗∗ <0.0001. NS, not significant. Western blot shown is a representative of three independent experiments. FC, fold change; NMD, nonsense-mediated decay; NMDi, NMD inhibitor.

**Figure 3:**
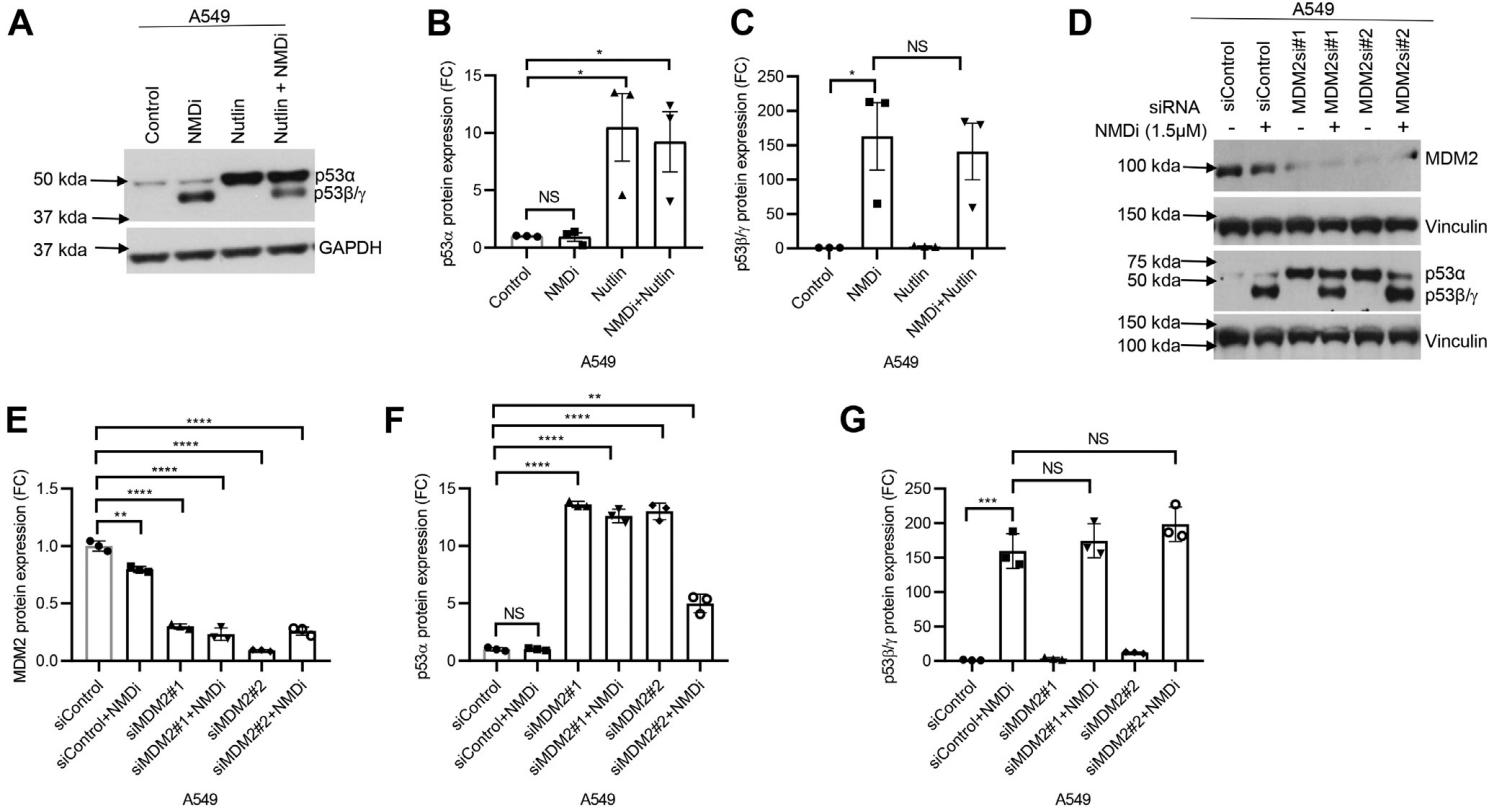
**p53β/γ isoforms are resistant to MDM2-mediated degradation.***A*, Western blot analysis showing p53α and p53β/γ expression in A549 cells treated with indicated drugs. *B* and *C*, quantification of p53α and p53β/γ protein expression, respectively, in A549 cells treated with the indicated drugs. *D*, Western blot analysis of A549 cells treated with indicated siRNAs and either dimethyl sulfoxide or nonsense-mediated decay inhibitor (NMDi). *E*–*G*, quantification of MDM2, p53α, and p53β/γ protein expression, respectively, in A549 cells treated with the indicated siRNAs and either dimethyl sulfoxide or NMDi. Western blot analysis quantifications shown (n = 3) are from three independent experiments. Mean ± SD, *p* values, two-tailed *t* tests, ∗ ≤0.05, ∗∗ <0.01, ∗∗∗ <0.001, ∗∗∗∗ <0.0001, NS, not significant. Western blots shown are a representative of three independent experiments. NMDi, nonsense-mediated decay inhibitor.

To confirm the identity of NMD inhibition-induced p53β and p53γ, we performed RNAi-mediated knockdown of total p53, p53 intron 9, p53β, and p53γ in A549 cells and treated the cells with NMDi. RNAi-mediated knockdown of total p53, p53 intron 9, p53β, and p53γ confirmed that p53 isoforms induced by NMD inhibition are indeed p53β and p53γ, derived from alternative splicing of intron 9 (Fig. 2, *A*–*D*). To test whether p53β and p53γ isoforms are susceptible to MDM2-mediated degradation, we treated A549 (Fig. 3*A*), H1944, and p53-null H1299 cells in which we overexpressed p53β and p53γ (Fig. S4*A*), with either NMDi or an MDM2-specific inhibitor nutlin (8) or both. Western blot analysis for p53 protein expression in these samples indicated a dramatic increase in the expression of p53α but not p53β and p53γ upon Nutlin treatment (Fig. 3, *A*–*C* and Fig. S4, *B* and *C*), indicating that only p53α but not p53β/γ is susceptible for MDM2-mediated degradation. To further confirm this result, we performed siRNA-mediated depletion of MDM2 in A549 cells and treated cells with either dimethyl sulfoxide (DMSO) or with NMDi (Fig. 3*D*). Western blot analysis indicated an increased expression of p53α but not p53β/γ in MDM2-depleted cells, whereas NMDi led to increased levels of p53β/γ but not p53α (Fig. 3, *D*–*G*). Taken together, these results indicate that NMD inhibition stabilizes alternatively spliced, MDM2-resistant p53β and p53γ isoforms.

**Figure 4:**
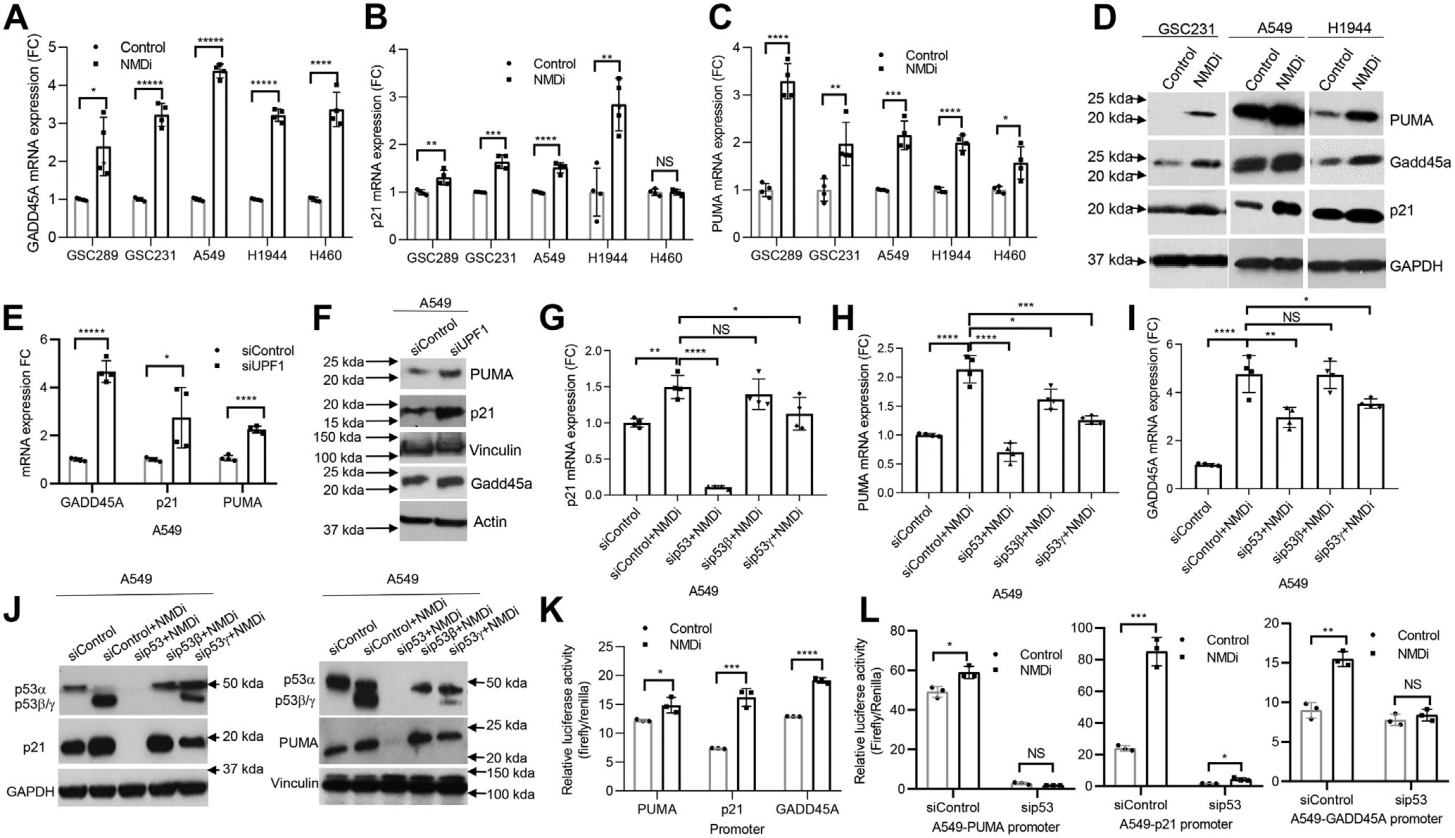
**NMD inhibition upregulates p53 transcriptional targets in a p53-dependent manner.***A*–*C*, FC in the mRNA expression of p53 transcriptional targets GADD45A, p21, and PUMA, respectively, upon NMDi treatment. *D*, Western blot showing increased PUMA, Gadd45a, and p21 expression in NMDi-treated cells (quantifications in Fig. S5). *E*, mRNA expression FC of GADD45A, p21, and PUMA in A549 treated with the indicated siRNAs. *F*, Western blot analysis showing increased PUMA, p21, and Gadd45a expression in A549 treated with indicated siRNAs (quantification in Fig. S5). *G*–*I*, mRNA expression FC of p53 transcriptional targets p21, GADD45A, and PUMA, respectively, upon NMD inhibition in A549 cells treated with the indicated siRNAs. *J*, Western blot analysis showing p21 (*left panel*) and PUMA (*right panel*) expression upon NMD inhibition in A549 cells treated with the siRNAs shown (quantification in Fig. S6). *K*, NMD inhibition increases binding to the promoters derived from p53 transcriptional targets PUMA, p21, and GADD45A. *L*, increased binding of NMDi-treated cells to the promoters of p53 transcriptional targets is p53 dependent. Luciferase activity is mean ± SD of three independent experiments. RT-qPCR analysis shown (n = 4) are at least from two independent experiments. Mean ± SD, *p* values, two-tailed *t* tests, ∗ ≤0.05, ∗∗ <0.01, ∗∗∗ <0.001, ∗∗∗∗ <0.0001, ∗∗∗∗∗ <0.00001, NS, not significant. FC, fold change, NMD, nonsense-mediated decay; NMDi, NMD inhibitor.

We next tested whether NMD inhibition resulted in p53 pathway activation, by assessing mRNA levels of p53 transcriptional targets GADD45A, p21, and PUMA. We observed a significant increase in the mRNA levels of GADD45A and PUMA following NMDi treatment in all five cell lines, whereas p21 mRNA was induced to a lesser extent in majority of them (Fig. 4, *A*–*C*), suggesting that inhibition of NMD activates p53-inducible promoters, although the extent of activation is either promoter dependent or cell type specific. Western blot analysis indicated an increased expression of these proteins in NMDi-treated GSC231, A549, and H1944 cells (Fig. 4*D* and Fig. S5*A*). We next examined whether UPF1-depleted cells also show similar p53 pathway activation as NMDi-treated cells. We observed that UPF1 depletion also increased GADD45A, p21, and PUMA mRNA and protein expression (Fig. 4, *E* and *F* and Fig. S5, *B* and *C*). These results are consistent with prior studies showing that p53 isoforms enhance the gene expression of p53α target genes (12).

To rule out the possibility that NMD inhibition-induced p53 pathway activation is a nonspecific effect of NMD inhibition, we performed mRNA and protein expression analysis of p53 transcriptional targets in p53-depleted cells treated with NMDi. Our results indicated that depletion of total p53 significantly reduced the NMDi-induced p21 and PUMA expression confirming that the observed increase in their expression upon NMD inhibition is p53 mediated (Fig. 4, *G* and *H*). Compared with p21 and PUMA, the extent of reduction in NMDi-induced GADD45A expression was less in p53-depleted cells (Fig. 4*I*), suggesting that NMD inhibition may also induce GADD45A by p53-independent mechanisms. Our data also showed that, among the two isoforms, p53γ knockdown had the greatest reduction in the expression of NMD inhibition-induced p53 transcriptional targets (Fig. 4, *G*–*J* and Fig. S6, *A* and *B*), suggesting that the relative contribution of p53γ is greater than that of p53β in NMD inhibition-induced p53 pathway activation. Alternatively, it is possible that the p53β and γ isoforms interact with other p53 isoforms in a collaborative manner and influence different p53-mediated pathways (25).

In order to test whether NMD inhibition-induced p53 pathway activation involves increased binding to p53 target promoters, we transfected the luciferase reporters containing *PUMA* (26), *p21*, and *GADD45A* promoter sequences with p53-binding sites and assessed the luciferase activity with or without inhibiting NMD in A549 cells. Our results demonstrated a significant increase in the luciferase activity in NMD-inhibited cells, indicating an increased binding of p53 to its transcriptional target promoters upon NMD inhibition (Fig. 4*K*). To confirm whether NMD inhibition-induced increase in p53-target-promoter binding is mediated by p53, we knocked down p53 in A549 cells and performed promoter binding assays with and without NMD inhibition (Fig. 4*L*). Our results indicated that NMDi-induced increase in promoter binding activity is indeed p53 dependent (Fig. 4*L*).

We next tested whether p53β and p53γ isoforms contribute in the enhanced binding to p53 transcriptional target promoters. To investigate this, we overexpressed p53β and p53γ in p53-null H1299 cells and assessed the cells for *PUMA*, *p21*, and *GADD45A* promoter binding activity by luciferase reporter assay (Fig. S7). The results indicated that, although cells overexpressing p53β significantly enhanced the luciferase activity for all three promoters, those that overexpressed p53γ showed only a modest increase for *p21* and *GADD45A* promoters (Fig. S7), suggesting that, in the absence of p53α, overexpression of p53γ alone is not sufficient to increase the expression of these p53 transcriptional targets. Alternatively, p53γ may only act in conjunction with other p53 isoforms to increase p53 transcriptional activity.

Taken together, our results indicate that NMD inhibition increases the transcription of p21 and PUMA in a p53-dependent manner and that p53β and p53γ promote this. Moreover, our results are in agreement with earlier findings indicating that p53 isoforms can regulate p53 transcriptional activity (12).

### NMD inhibition restores the p53 pathway and triggers p53β/γ expression in p53 mutant cancer cells

**Figure 5:**
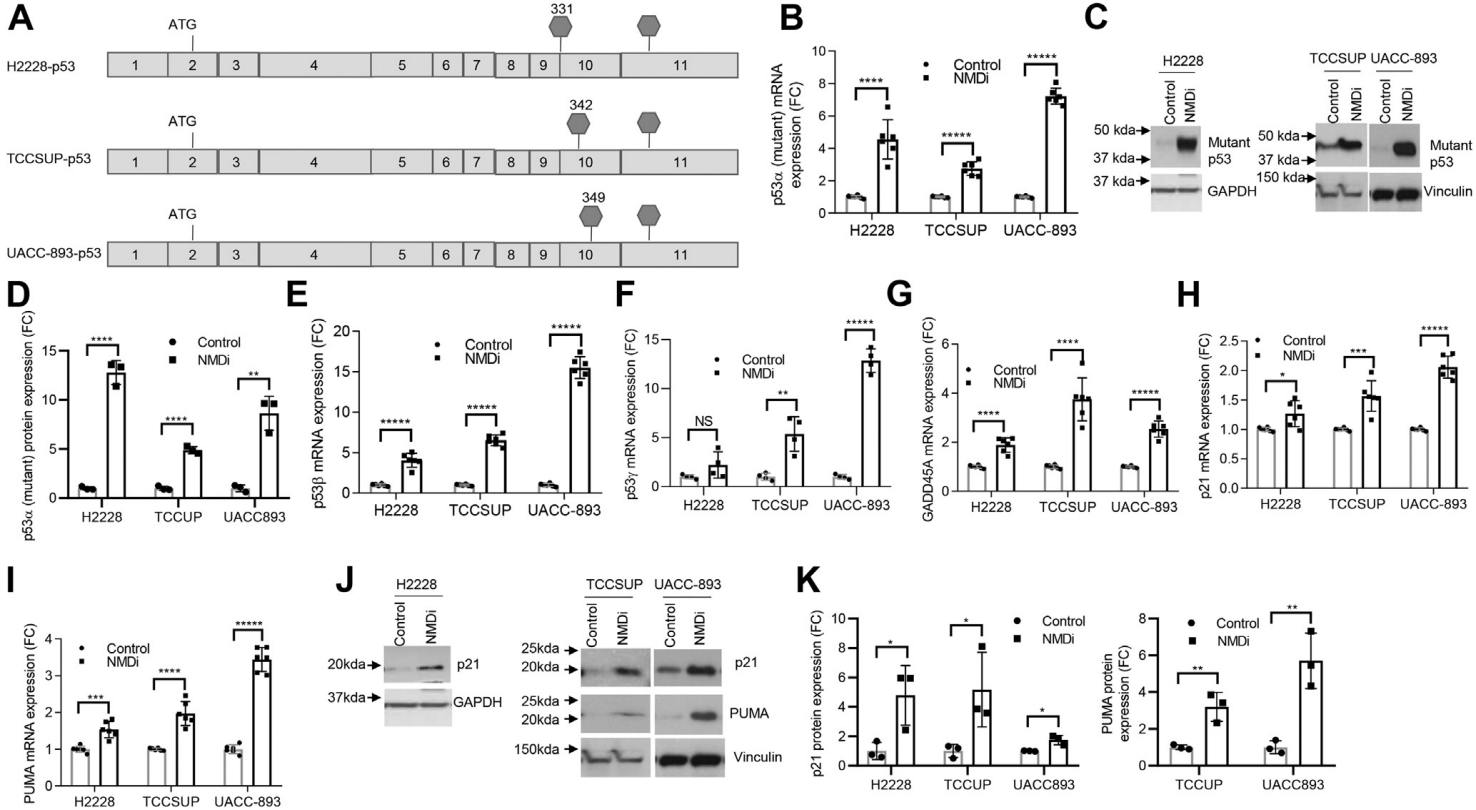
**NMD inhibition restores the p53 pathway and triggers p53β/γ expression in p53 mutant cancer cells.***A*, schematic showing the location of premature termination codons in p53 mutant cell lines. *B*, mRNA expression FC of mutant p53α in cells treated with NMDi. *C*, Western blots showing increased expression of truncated p53 upon NMDi treatment in p53 mutant cells. *D*, Western blot quantification of mutant p53 protein expression shown in (*C*), in indicated cells treated with NMDi (*E* and *F*) mRNA expression FC of p53β and p53γ isoforms, respectively, in NMDi-treated p53 mutant cells. *G*–*I*, mRNA expression FC of p53 transcriptional targets GADD45A, p21, and PUMA, respectively, upon NMDi treatment. *J*, Western blots showing increased expression of p21 and PUMA in NMDi-treated cell lines shown. *K*, Western blot quantification of p21 and PUMA protein expression in indicated cells treated with NMDi. GAPDH or vinculin were used as loading controls for Western blots. For H2228, Western blot from (*C*) was reprobed with p21 in (*J*). For TCCSUP and UACC-893, two sets of same samples were run side by side on the same gel; one set was probed with p53 and PUMA, another set was probed with p21 and Vinculin. Cells in *B*–*J* were treated with either dimethyl sulfoxide (Control) or 1 μM NMDi for 16 h. RT-qPCR data shown (n = 4–6) and Western blot quantifications are from three independent experiments. Mean ± SD, *p* values, two-tailed *t* tests, ∗ ≤0.05, ∗∗ <0.01, ∗∗∗ <0.001, ∗∗∗∗ <0.0001, ∗∗∗∗∗ <0.00001, NS, not significant. FC, fold change, NMD, nonsense-mediated decay; NMDi, NMD inhibitor.

Somatic mutations in *TP53* are found in about 45% of all cancers, and of these, 34% are truncating mutations (Table S3) that lead to p53 deficiency. About 4.8% of the truncating mutations are located either at the end of exon 9 (codon 331) or downstream of it (Table S3). If p53 transcripts bearing PTC-generating mutations at the C-terminal end are protected from degradation by NMD, they could produce near-full-length protein and potentially rescue some or all of p53 function (Fig. S8, *A* and *B*) in addition to the stabilization of p53β/γ isoforms, which would be unaffected by the downstream mutation. To test this, we utilized H2228 (NSCLC), TCCSUP (urinary bladder cancer), and UACC-893 (breast cancer) cells harboring PTCs in p53 exons 9 (H2228) and 10 (TCCSUP and UACC-893), respectively (Fig. 5*A*). NMDi treatment in these cell lines increased the expression of mutant p53α mRNA and protein (Fig. 5, *B*–*D*), consistent with the earlier studies reporting the rescue of nonsense mutation–bearing p53 transcripts by NMD inhibition (27, 28). In addition, NMDi increased the expression of p53β/γ transcripts (Fig. 5, *E* and *F*) and significantly increased mRNA and protein levels of p53 transcriptional targets (Fig. 5, *G*–*K*). These results indicate that, for *TP53* mutations resulting in PTCs in C-terminal exons, NMD inhibition may help restore p53 function by two distinct mechanisms: enabling the synthesis of near-full-length p53 protein by preventing the degradation of the mutant transcript and enhancing the expression of functional p53β/γ isoforms, which are truncated upstream of the PTC-inducing mutation. Moreover, in the case of cancers bearing mutations with loss-of-function or gain-of-function attributes downstream of p53 exon 9 (codon 331) (Table S3), NMD inhibition could restore p53 function by upregulating p53β/γ isoforms that lack the canonical C terminus and hence the mutations (Fig. S8, *C* and *D*).

### NMD inhibition induces apoptosis, reduces tumor cell viability, and enhances radiosensitivity

**Figure 6:**
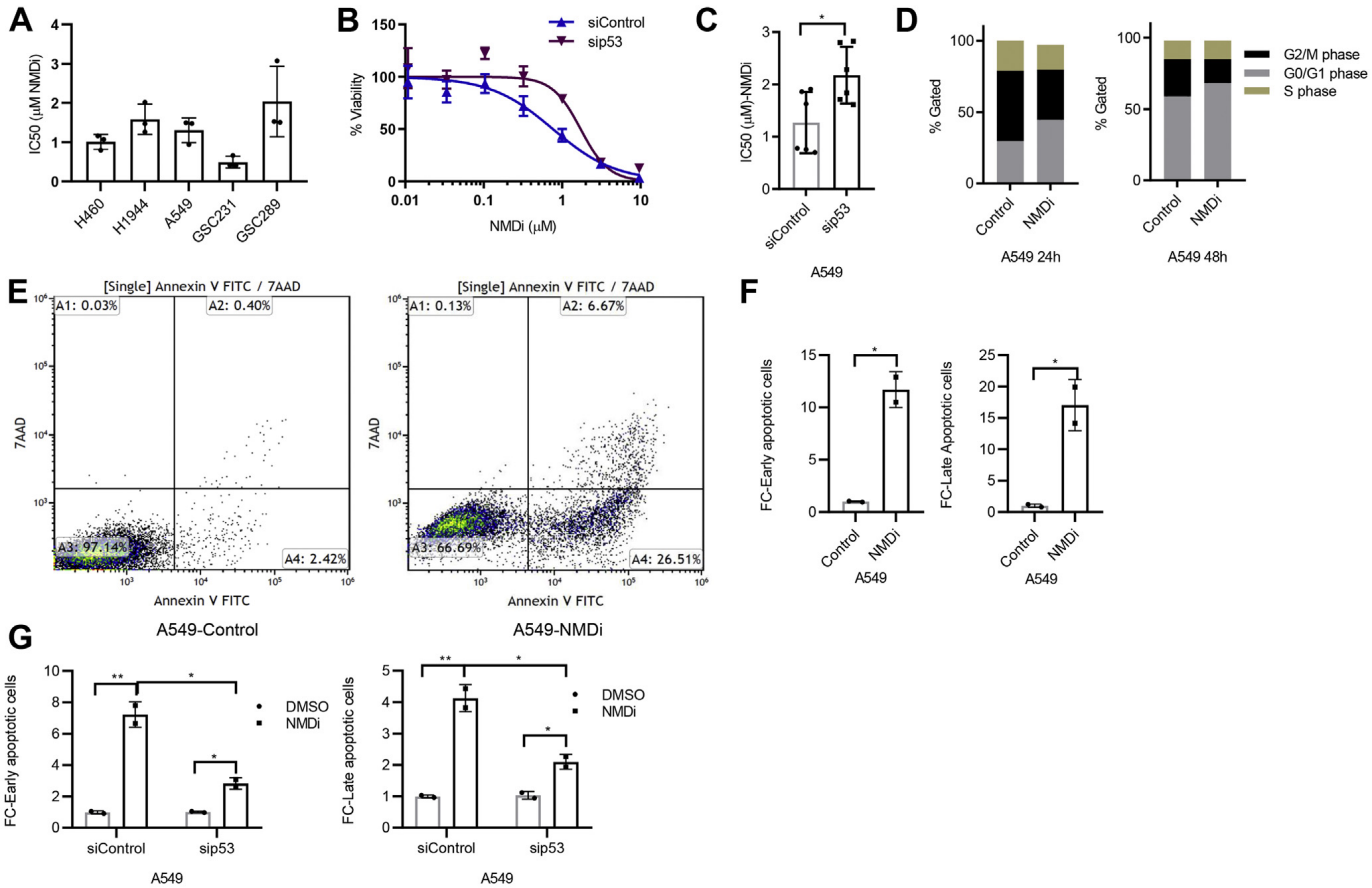
**p53 contributes to NMDi-induced cell death and apoptosis.***A*, NMDi treatment reduces viability of MDM2-overexpressing NSCLC and GBM cell lines. Cells were treated with DMSO or seven different concentrations of serially 3-fold-diluted NMDi, highest concentration being 9.6 μM NMDi, for 72 h. IC_50_ calculations were done as described in Experimental procedures. *B*, p53 depletion reduces sensitivity to NMDi and *C*, increases IC_50_ in A549 cells. *D*, NMDi disrupts cell cycle progression. A549 cells were treated with either DMSO or NMDi (1 μM) for the indicated time points, fixed in 70% ethanol, stained with propidium iodide, and analyzed by flow cytometry as described in Experimental procedures. *E*, fluorescence-activated cell sorting analysis of either control or NMDi-treated A549 cells. *Numbers* in quadrant A2 indicate percent of cells that have undergone apoptosis, and the *numbers* in quadrant A4 indicate percent of cells in early apoptotic phase. Data shown are the representatives of two independent experiments. *F*, FC in early and late apoptotic cells treated with NMDi, as compared with the DMSO-treated controls. *G*, p53 depletion reduces NMDi-induced apoptosis. Scatter plots represent FC in early and late apoptotic cells in A549 cells treated with the indicated siRNAs and either DMSO or NMDi. Data shown are the average of two independent experiments. Mean ± SD, *p* values, two-tailed *t* tests, ∗ ≤0.05, ∗∗ <0.01. DMSO, dimethyl sulfoxide; FC, fold change; NMDi, NMD inhibitor.

P53 activation confers radiosensitivity and chemosensitivity in cancer cells (29, 30). Our finding that NMD inhibition reactivated the p53 pathway prompted us to evaluate whether NMDi reduces cell viability and enhances radiation sensitivity. Our results indicated that NMDi treatment reduces the viability of both NSCLC and GBM cells with an IC_50_ (dose of NMDi at which 50% of the cells die) value ranging from 0.4 to 3.0 μM (Fig. 6*A*), compared with their untreated controls. To test whether p53 plays a role in NMDi sensitivity, we depleted p53 in A549 cells and treated the cells with NMDi (Fig. 6, *B* and *C*). Our results showed a significantly decreased sensitivity to NMDi in the absence of p53, indicating that p53 contributes to NMDi sensitivity in these cells (Fig. 6, *B* and *C*).

Given that Puma and p21 are involved in apoptosis and cell cycle arrest, respectively, and were upregulated by NMD inhibition, we also performed cell cycle analysis and apoptosis assay. Cell cycle analysis showed increased G0/G1 and decreased G2/M populations in NMDi-treated cells, indicating that NMD inhibition disrupts cell cycle progression (Fig. 6*D*). Analysis of NMDi-treated cells for apoptosis by Annexin V FITC/7AAD staining indicated a significant increase in both early and late apoptotic cell population as compared with their respective controls (Fig. 6, *E* and *F*). To test whether p53 contributes for NMDi-induced apoptosis, we depleted p53 in A549, treated cells with NMDi, and performed apoptosis assay in these cells (Fig. 6*G* and Fig. S9). Our results showed a significant decrease in NMD inhibition-induced apoptosis in p53-depleted cells compared with the controls (Fig. 6*G* and Fig. S9).

**Figure 7:**
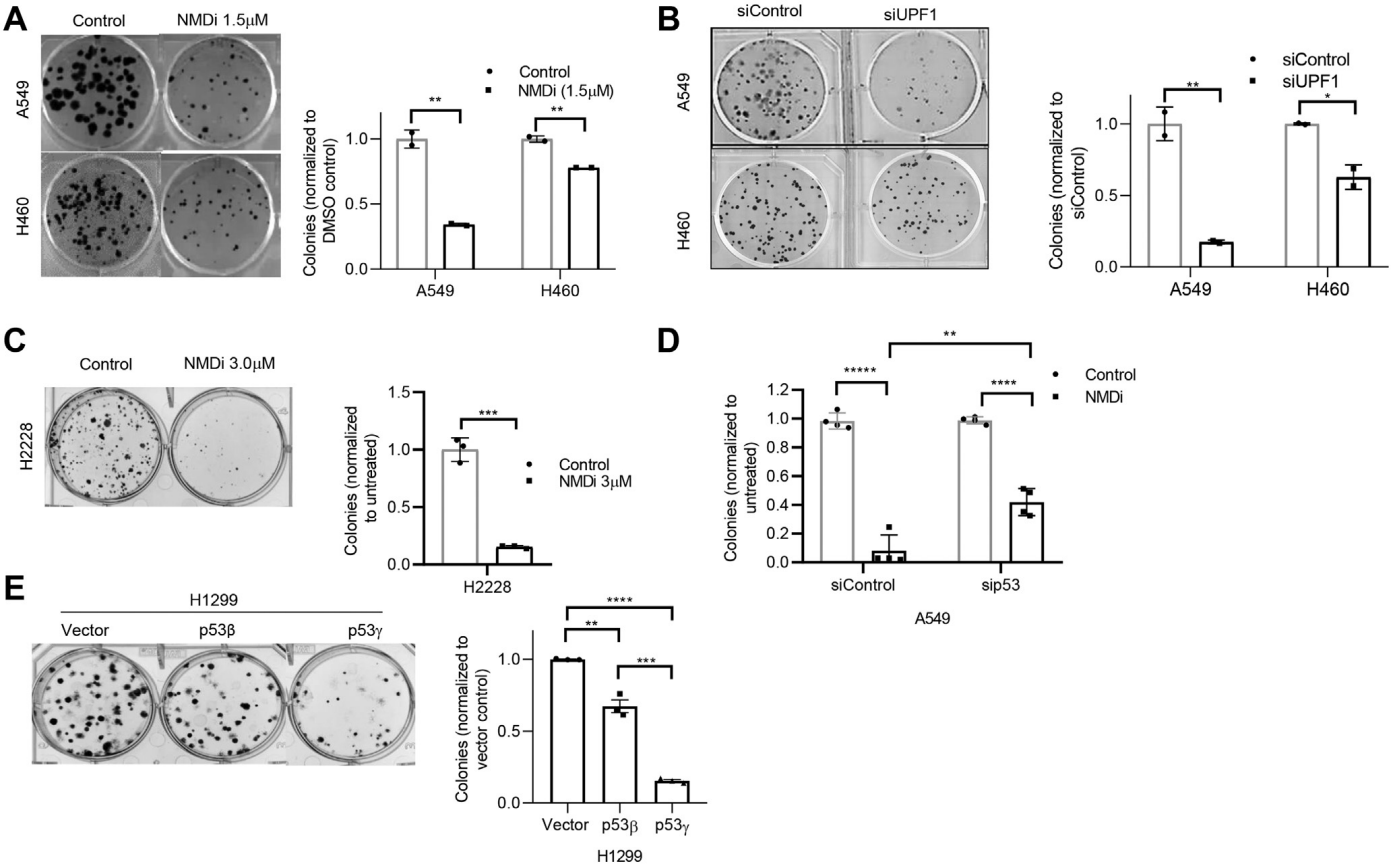
**NMD inhibition reduces colony-forming ability.***A*–*C*, clonogenic assay showing reduction in the colony-forming ability upon NMD inhibition either by NMDi treatment (*A* and *C*) or by UPF1 depletion (*B*) in the cell lines shown. *Left panels* show the colonies and *right panels* show the quantification (n = 2 experiments for *A* and *B* and n = 3 for *C*) of the colonies from the clonogenic assay. Cells were treated with either DMSO or indicated concentrations of NMDi. *D*, quantification of clonogenic assay (n = 4) performed on A549 cells treated with either siControl or sip53 and then with NMDi. *E*, clonogenic assay (*left panel*) of H1299 cells overexpressing the indicated p53 isoforms. Quantification (*right panel*) of the data shown in the *left panel* (n = 3). Mean ± SD, *p* values, two-tailed *t* tests, ∗ ≤0.05, ∗∗ <0.01, ∗∗∗ <0.001, ∗∗∗∗ <0.0001, ∗∗∗∗∗ <0.00001. DMSO, dimethyl sulfoxide; NMD, nonsense-mediated decay; NMDi, NMD inhibitor.

We next evaluated the colony-forming ability of NMDi-treated NSCLC cells. Our assessment showed that NMDi treatment significantly reduced the colony-forming ability of A549 and H460 cells (Fig. 7*A*). UPF1 depletion showed similar results (Fig. 7*B*), further supporting the finding. Given that NMD inhibition increased the expression of near-full-length p53 protein, induced p53β/γ, and activated p53 pathway in cells harboring p53 mutations, we next tested whether NMD inhibition reduces colony-forming ability in these cells also, by treating H2228 cells with NMDi and found that, as in the case of cells harboring WT p53 (A549 and H460), NMDi treatment reduced the colony-forming ability of p53 mutant H2228 cells also (Fig. 7*C*).

To examine whether reduction in the colony-forming ability of NMDi treated cells is because of p53 pathway activation, we treated p53-depleted A549 cells with NMDi and assessed the cells for colony-forming ability. Our data showed that p53 depletion partially but significantly restored NMDi-induced reduction in colony-forming ability (Fig. 7*D*). We then tested whether p53β/γ contribute to colony reduction by assessing the colony-forming ability of H1299 cells overexpressing p53β and p53γ. Our results showed significantly fewer colonies in both p53β- and p53γ-overexpressing cells compared with the vector control (Fig. 7*E*). Of interest, even though H1299 cells overexpressing p53β showed higher p53 target-promoter-binding activity (Fig. S7), cells overexpressing p53γ formed considerably fewer colonies than those overexpressing p53β (Fig. 7*E*). This may be because p53β and p53γ affect p53-mediated cellular responses *via* distinct mechanisms and may play a role in different biological functions (15).

**Figure 8:**
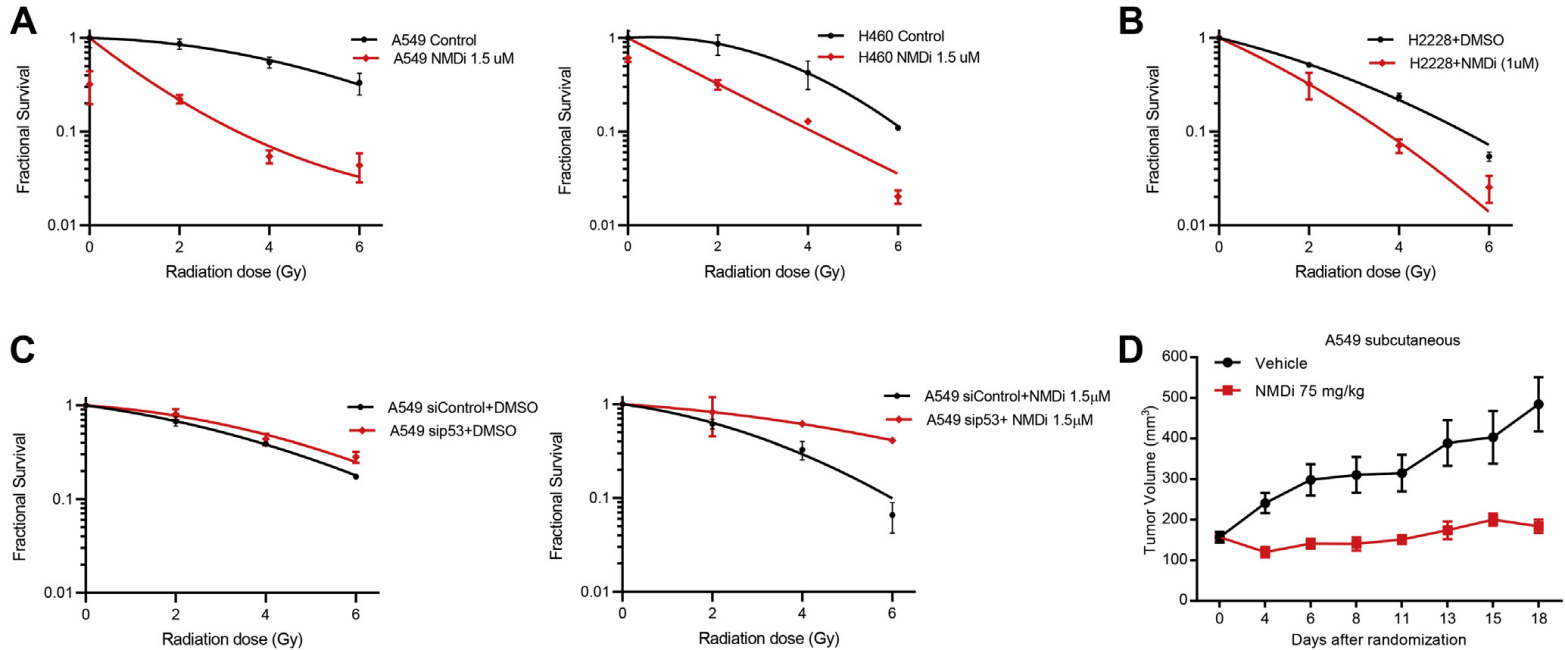
**NMD inhibition increases radiation sensitivity and impairs tumor growth.***A*, NMD inhibition increases radiation sensitivity in A549, H460, and p53 mutant H2228 cells (*B*). *C*, p53 depletion attenuates NMDi-induced radiation sensitivity. Radiation sensitivity of A549 cells treated with the indicated siRNAs and either DMSO (*left panel*) or NMDi (*right panel*). Clonogenic survival assay and fractional survival analysis were done as described in Experimental procedures. *D*, subcutaneous xenograft tumor growth in nude mice injected with A549 cells and treated with either vehicle or NMDi. Mice injected with A549 cells were randomized and treated (n = 10 per group) as described in Experimental procedures. DMSO, dimethyl sulfoxide; NMD, nonsense-mediated decay; NMDi, NMD inhibitor.

Since p53 is also implied in increasing the response to ionizing radiation (IR) (29), we next treated A549, H460, and p53 mutant H2228 cells with radiation alone or in combination with NMDi and evaluated cell viability. NMD inhibition increased the radiotherapy sensitivity of both p53WT (A549 and H460) and p53 mutant cancer cells (Fig. 8, *A* and *B*). To test whether NMD inhibition-induced radiation sensitivity is p53 mediated, we treated p53-depleted A549 cells with NMDi and assessed radiation sensitivity in these cells. Our data showed that depletion of p53 considerably reduced NMDi-induced radiation sensitivity (Fig. 8*C*), indicating that p53 plays a role in enhancing radiation sensitivity. We then asked whether p53β and p53γ aid in increasing radiation sensitivity. To test this, we overexpressed p53β and p53γ in A549 cells and analyzed their radiation sensitivity in the absence of NMD inhibition (Fig. S10, *A*–*C*). Our results showed a modest increase in the radiation sensitivity in the cells that overexpressed p53γ (Fig. S10*C*), suggesting that, in the absence of NMD inhibition, overexpression of either p53β or p53γ alone is not sufficient to increase the radiation sensitivity. Collectively, our results indicate that NMD inhibition increases p53β and p53γ expression, activates p53 pathway, induces apoptosis, disrupts cell cycle progression leading to reduction in cell viability and colony-forming ability, and sensitizes cells to radiation. Furthermore, our data show that the effects of NMD inhibition on apoptosis, cell viability, tumor colony growth, and radiosensitivity are at least in part p53 dependent, although we cannot rule out the possibility that other NMD-regulated factors contribute to the effects as well.

### NMD inhibition impairs tumor growth

We next evaluated the therapeutic implications of NMD inhibition by assessing the *in vivo* growth of A549 tumors. Nude mice were injected subcutaneously with A549 cells and randomized to receive vehicle or NMDi. In this tumor model, we observed a significant reduction in tumor volume in NMDi-treated animals, as compared with the vehicle-treated animals (Fig. 8*D*). To investigate whether NMDi treatment induced the expression of p53β/γ isoforms and activated p53 pathway *in vivo*, we collected tumor tissues from animals and evaluated the expression of p53β/γ and p53 transcriptional targets by RT-qPCR. We observed a significant increase in the mRNA expression of p53γ and PUMA and an increasing trend in the expression of p53β and GADD45A in NMDi-treated tumors compared with their respective vehicle-treated controls (Fig. S11). These findings indicate that NMD inhibition impairs tumor growth, which may be partly due to p53 pathway activation.

**Figure 9:**
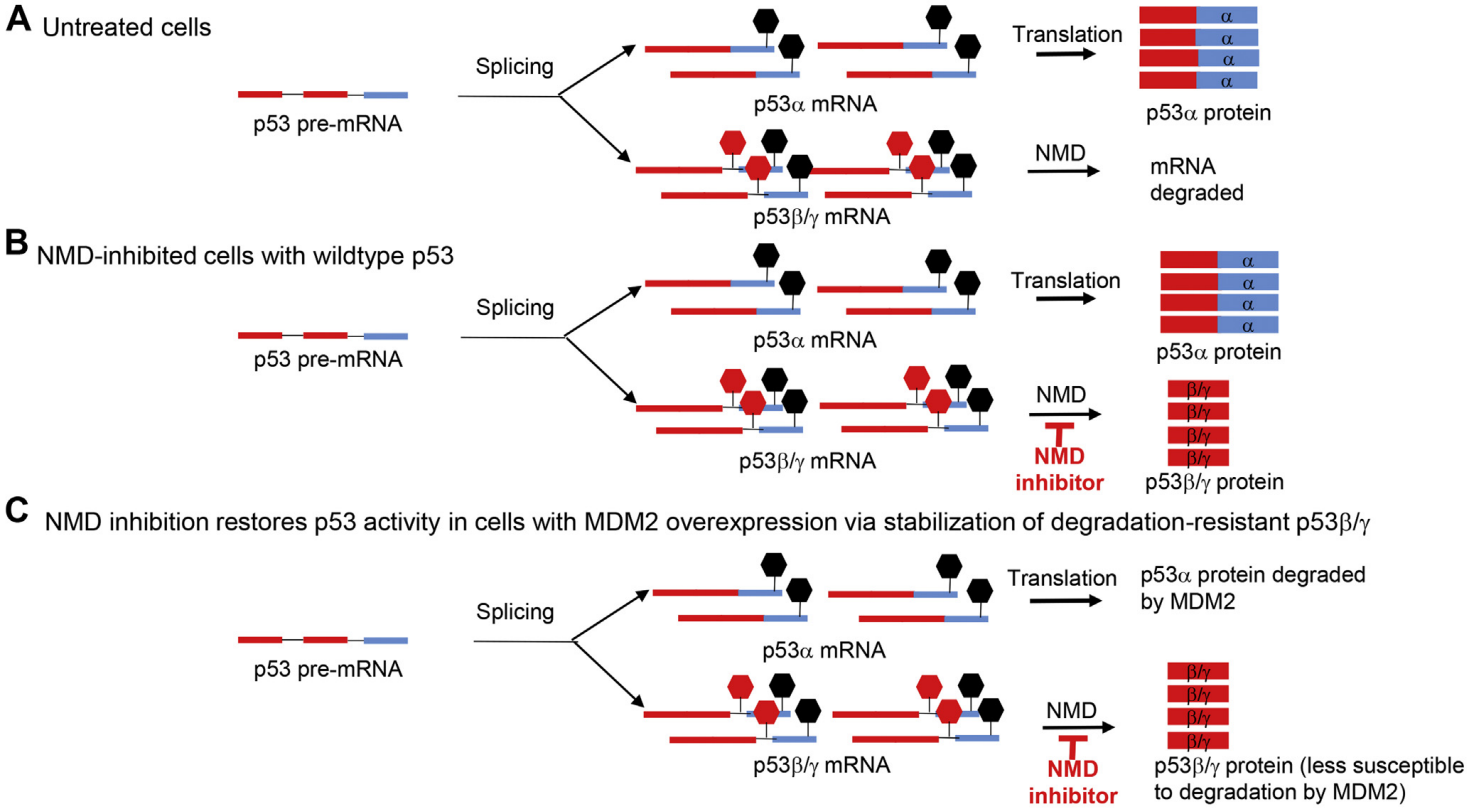
**Model depicting the p53 restoration strategy in p53-deficient tumors bearing MDM2 amplification/overexpression.***A*, in normal cells, p53 pre-mRNA splicing results in p53α and p53β/γ mRNAs. P53β/γ mRNA is degraded by NMD; p53α mRNA is translated. *B*, NMD inhibition stabilizes p53β/γ mRNA, resulting in the expression of p53β/γ protein. *C*, in MDM2-amplified cells, p53α protein is degraded. NMD inhibition overcomes p53 deficiency by increasing the expression of degradation-resistant p53β/γ that lack the C-terminal MDM2-binding sites. Stop sign in *black*, normal stop codon; stop sign in *red*, premature termination codon. P53 exons 1 to 9/i9 are depicted in *red*; exons 10 and 11 are depicted in *light blue*. NMD, nonsense-mediated decay.

Taken together, our data provide evidence that NMD inhibition stabilizes p53β and p53γ isoforms and restores p53 activity in tumors with p53 deficiency arising from either inactivating mutations downstream of exon 9 (Fig. S8) or due to MDM2 overexpression (Fig. 9, *A*–*C*). Since p53β/γ isoforms contribute to NMD inhibition-induced p53 pathway activation and are less susceptible to MDM2-mediated degradation than p53α, NMD inhibition may particularly benefit cancers that are caused by MDM2-mediated degradation of WT p53 (Fig. 9*C*).

## Discussion

P53 is the most frequently inactivated tumor suppressor in cancer. Here, we investigated NMD inhibition as a strategy to reactivate p53 and, in turn, impair tumor growth and sensitize tumor cells to commonly used therapeutic regimens. We provide evidence that NMD inhibition stabilizes the p53β and γ isoforms and restores p53 activity in not only p53-mutant cancer cells but also in cancer cells expressing WT p53 but are p53 deficient because of overexpression of its negative regulator MDM2.

NMD inhibition using aminoglycosides or ataluren (PTC124) has been used as a therapeutic strategy in many diseases caused by nonsense mutations including Duchenne muscular dystrophy and cystic fibrosis (31, 32). These pharmacological NMD inhibitors act by suppressing PTCs caused by nonsense mutations but are not effective at protecting the degradation of physiological NMD substrates such as alternatively spliced transcripts. By contrast, the pyrimidine derivative NMDi (compound 11j in reference 22) (22) used in the current study not only efficiently reverses the NMD of the PTC-bearing p53 mRNA transcripts but also physiological NMD substrates such as p53β and p53γ, derived from nonmutant gene. NMDi was shown to be highly specific for hSMG-1 kinase as compared with 27 other kinases. NMDi inhibited NMD by disrupting hSMG1 kinase–mediated phosphorylation of the key NMD factor UPF1, a critical step required for degradation of NMD substrate-mRNAs in humans (11, 22). In our studies, apart from enabling the synthesis of near-full-length, functional p53 proteins in p53-mutant cancer cell lines, NMDi also protected p53β and p53γ from degradation. This can be advantageous in cancers bearing nonsense mutations downstream of p53 exon 9 as p53β and p53γ lack C terminus and hence mutations.

One important NMD-triggering feature is the location of a PTC with respect to the last exon–exon junction of an mRNA. In humans, PTCs located at least 50 to 55 nucleotides (nt) upstream of the last exon–exon junction trigger NMD (33, 34). Even though PTCs generated by alternative splicing of p53 intron 9 in exon 9β and 9γ of p53β and p53γ are 207 and 119 nt upstream, respectively, of the last exon–exon junction (p53 exon 10–exon 11 junction), earlier studies were not able to detect p53γ induction upon NMD inhibition and showed only p53β as an NMD substrate (19, 21). In this study, we show that NMD inhibition either genetically or pharmacologically reverses the degradation of both p53β and p53γ. mRNA decay analysis further confirmed the NMD susceptibility of both of these isoforms. NMD inhibition did not increase p53α mRNA expression, indicating that p53α is not an NMD substrate. On the contrary, NMD inhibition reduced the amount of p53 alpha. The mechanism underlying this reduction is not clear but merits further investigation.

The p53β and γ isoforms promote transcription of p53 target genes and retain tumor suppressive functions. For instance, p53β and p53γ were shown to promote apoptosis and senescence by inducing p53α-dependent transactivation of Bax and p21, respectively (12, 14, 15). Our study using PUMA, p21, and GADD45A reporter constructs harboring p53-binding sites showed increased luciferase activity upon NMD inhibition in a p53-dependent manner, suggesting that increased p53 pathway activation may be due to an increase in the transcriptional activity of p53. In addition, we also showed that overexpression of p53β, and to a lesser extent p53γ, in p53-null H1299 cells led to increased binding to PUMA, p21, and GADD45A promoters. These findings suggest that the p53β/γ isoforms may induce transcription of p53 target genes in both a p53α-dependent and independent manner. Consistent with this, it has been shown that p53β can induce apoptosis in a p53-independent manner, although to a lesser extent, compared with p53α (12). In patients with breast cancer, p53β and p53γ levels are associated with more favorable clinical outcome (35, 36). P53β expression showed a negative correlation with tumor size and positive correlation with survival in patients with breast cancer. High levels of p53β were particularly protective in patients with mutant p53 and may play a role in counteracting the damage inflicted by mutant p53 (36). Our results from overexpression of p53γ in p53-null H1299 cells indicated that, although expression of p53γ alone could increase PUMA promoter binding, it was not sufficient to increase the binding to p21 and GADD45A promoters. These findings suggest that p53γ might act in conjunction with p53α or other p53 isoforms in a promoter-specific manner to enhance the expression of p53 transcriptional targets. In line with this, patients with breast cancer expressing p53γ along with mutant p53 had a similar prognosis as those with WT-p53, whereas those who were devoid of p53γ and expressed only mutant p53 showed markedly poor prognosis (35). Taken together, our finding that NMD inhibition protects both p53β and p53γ isoforms from degradation even in p53 mutant cells signifies the therapeutic benefits of NMD inhibition in p53 mutant cancers.

We found that NMD inhibition sensitized NSCLC cells bearing either WT p53 or mutant p53 to IR. IR has been shown to inhibit SMG1 and trigger the expression of p53β, which in turn contributes to IR-induced cellular senescence in MCF-7 cells (37). In these cells, UPF1 knockdown did not induce p53β expression, and hence the authors concluded that the increased p53β expression was not because of NMD inhibition but due to enhanced IR-induced alternative splicing of p53 *via* the “SMG1-RPL26-SRSF7” pathway (37). By contrast, in our study, we found that knocking down UPF1 expression genetically or inhibiting SMG1 pharmacologically, both of which inhibit NMD, induced p53β even in the absence of IR in A549 and H460 cell lines (Fig. 1, Figs. S2 and S3). This, together with our mRNA decay analysis (Fig. S2), indicated that p53β is NMD susceptible and can be stabilized by inhibiting NMD. Moreover, we found that, in the absence of NMD inhibition, overexpression of p53β or p53γ alone was not sufficient to increase the radiation sensitivity in A549 cells. However, we cannot rule out the possibility that other NMD-regulated factors contribute to the antitumor effects elicited by NMD inhibition as our data show that the effects of NMD inhibition on radiosensitivity, apoptosis, cell viability, and tumor colony growth are partly p53 dependent.

P53 deficiency is the most common alteration observed across cancers, and p53 reactivation strategies therefore remain a challenge of great clinical significance. Drugs developed to target a particular p53 mutation have limited usage against other p53 mutations. For example, NSC31397 restores p53 activity in R175H-p53 mutant cancer cells and p53R3 restores DNA binding of R175H and R273H p53 mutants (38). By contrast, the approach we describe here for p53 restoration could potentially be broadly applied to not only tumors with p53 mutations downstream of exon 9 but also to those in which WT p53 is degraded by negative regulators such as MDM2. As the p53β/γ isoforms lack the canonical C terminus, this novel approach could also be used to eliminate loss-of-function or gain-of-function mutations in 2.7% of all cancers bearing these mutations downstream of p53 exon 9 (Table S3 and Fig. S8). Collectively, the NMD inhibition strategy could benefit approximately 6% of all cancers, which bear the appropriate p53 mutations and MDM2 amplification (MDM2 amplified, 3.7%; and mutations downstream of exon 9, 2.7%: Tables S1 and S3). Our findings showing that the p53β/γ isoforms are MDM2 resistant and that NMD inhibition restores p53 pathway, increases radiation sensitivity, reduces tumor cell colony forming ability, and impairs tumor growth in MDM2 overexpressing tumor cells signify the clinical relevance of NMD inhibition for this particular subgroup and also other smaller p53-deficient subgroups (*e.g.*, p53 mutations downstream of exon 9) for which this approach could be used.

## Experimental procedures

### Cell lines

A549, H460, H1944, H2228, TCCSUP, and UACC-893 cells were from American Type Culture Collection. GSC231 and GSC289 were generated and finger printed at the laboratories of Dr Erik Sulman and Dr Frederick Lang Jr (Department of Neurosurgery, UT-M.D. Anderson Cancer Center). A549, H460, and H2228 were grown in RPMI with 10% fetal bovine serum (FBS). TCCSUP grown in MEM with 10% FBS, 1% NEAA, and 1 mM sodium pyruvate. UACC-893 cells were grown in Lebovitz’s L-15 Medium (American Type Culture Collection) with 10% FBS. GSC231 and GSC289 cells were grown in Neutral Basal Medium (DMEM/F12 50/50, 1× B27 supplement, 20 ng/ml EGF, 20 ng/ml bFGF, 1% Pen/Strep solution). All cell lines were negative for mycoplasma.

### NMDi treatment

NMDi was supplied by Dr Phillip Jones (Institute for Applied Cancer Science, UT-MD Anderson Cancer Center). To assess the expression of p53β/γ and p53 transcriptional targets upon NMD inhibition, cells were treated with either DMSO or NMDi (1.0 μM for H460, 1.5 μM for A549, 2.0 μM for H1944 and GSC231, and 3.0 μM for GSC289) for ∼16 h and harvested for preparing RNA or protein lysates.

### MDM2 inhibition

A549 and H1944 cells were treated with either NMDi (1.5 and 2.0 μM, respectively) or nutlin 3 (5 and 10 μM, respectively) (Catalog# S1061, Selleck Chemicals LLC) or with a combination of both for simultaneous inhibition of MDM2 and NMD for ∼16 h and protein lysates prepared. H1299 cells overexpressing p53β and p53γ were treated with 10 μM Nutlin 3.

### Quantitative PCR

RNA was extracted by using either TRI Reagent Soln (Ambion) or RNeasy kit (Qiagen), according to manufacturer’s instructions. Total RNA was quantified using NANO DROP 2000C spectrophotometer (ThermoScientific). cDNA was synthesized by reverse transcription using iScriptReverse Transcription Supermix for RT-qPCR (BIO-RAD), using 1 μg of total RNA, according to manufacturer’s instructions. Quantitative PCR (qPCR) was done on cDNA using PerfeCta SYBR Green FastMix Low Rox probes (Quantabio) and the primers listed in Table S2. Real-Time PCR was done in 7500 Fast Real-Time PCR System (Applied Biosystems). The ΔΔ*C*t method was used to calculate fold changes in expression. Human GAPDH was used as an endogenous control for mRNA expression. Fold changes expressed are normalized either to the siControl or DMSO-treated controls.

### Western blot

Protein lysates (30–40 μg) were resolved on 4% to 15% gradient SDS-PAGE gels and transferred to polyvinylidene difluoride (PVDF) membrane using a wet system (BIO-RAD). Membranes were washed briefly in 1× TBS-Tween (TBST) and blocked in 5% (W/V) dried milk in TBST (1 h), incubated overnight at 4 °C in primary antibodies diluted 1:1000 in 5% (W/V) BSA-containing TBST, washed three times in TBST and incubated in appropriate horseradish peroxidase–conjugated secondary antibody. Membranes were developed using Super Signal West Pico PLUS Chemiluminescent Substrate (Thermo Scientific) and exposed to Blue Lite Autorad Film (GeneMate). GAPDH, actin, or vinculin was used as loading control. Antibodies used were p53 (7F5, Catalog # 2527S), PUMA (D30C10, Catalog #12450S), p21 Waf1/Cip1 (DCS60, Catalog # 2946S), Gadd45a (Catalog # 4632S), Upf1 (Catalog # 9435), GAPDH (Catalog # 5174) from Cell Signaling Technology, MDM2 (Catalog # sc-56154) from Santa Cruz Biotechnology, and β-actin (Catalog # A5441) and Vinculin (Catalog #V9131) from SIGMA. Protein expressions were normalized to that of GAPDH, β-actin, or vinculin.

### Assessment of mRNA decay

To assess mRNA decay of p53 isoforms, A549 cells were treated with either DMSO or NMDi (1 μM) for 16 h; transcription was then inhibited by adding 5,6-Dichloro-1-β-D-ribofuranosylbenzimidazole (100 μM), and RNA was extracted at various time points. mRNA expression analysis was performed by RT-qPCR.

### RNAi-mediated knockdown

siRNAs against human UPF1 (aagatgcagttccgctccatttt) (39), p53β (ggaccagaccagctttc), p53γ (cccttcagatgctacttga), and p53 intron 9 (gaugcuacuugacuuacga) (13), total p53 (gagguuggcucugacugua, siRNA ID: SASI_Hs02_00302766), and MDM2 (siRNA ID: SASI_Hs02_00132044 and SASI_Hs02_00132038) were purchased from Sigma-Aldrich. About 700,000 to 1 million cells were plated in 100-mm tissue culture plates overnight. siRNA stocks (100 μM) were diluted in RNase-free water, mixed with DharmaFECT 1 (Dharmacon), incubated for 20 min at room temperature, and added to the cells in medium lacking penicillin/streptomycin, at a final concentration of 100 nM siRNA in a total volume of 8 ml/plate. Twenty-four hours post transfection, cells were split into two 60-mm plates each, for protein and RNA extraction. siUPF1-treated cells were harvested 72 h post transfection. For siMDM2, sip53, sip53 intron 9, sip53β, and sip53γ-treated cells, NMDi was added 56 h post transfection to a final concentration of 1.5 μM and cells were harvested 16 h post NMDi treatment for protein and RNA extraction.

### Luciferase reporter assay

Cells (50,000/well) were plated in 24-well plates and transfected with 1 μg/well of reporter constructs containing PUMA (26) (4XBS2 WT Catalog # 16593, Addgene), p21 (PG13-Luc, Catalog # 16442, Addgene), or GADD45A (GADD45 WT-Luc, Catalog # 8356, Addgene); promoters harboring p53-binding sites were transfected using Lipofectamine 2000 (Invitrogen). Renilla construct (25 ng/well, Promega) was cotransfected as a control for transfection efficiency. Approximately 24 h post transfection, A549 cells were treated with 2.0 μM NMDi for 17 h. Luciferase assay was performed using Dual-Luciferase Assay System (Catalog # E1960, Promega) according to manufacturer’s instructions. Luminescence was measured using GLOMAX 20/20 Luminometer (Promega). Luciferase activity was normalized with Renilla activity.

For assessing promoter-binding ability of p53-depleted A549 cells, cells transfected with either siControl or sip53 (50,000 cells/well) were used and the experiment was performed as described above.

### p53β and p53γ overexpression

H1299 cells were either transiently or stably transfected with 5 μg of either p53as-i9 (vector control containing p53 intron 9 cloned in the antisense direction) or plasmids containing p53β or p53γ, using Lipofectamine 2000K (Invitrogen). For stable transfection in H1299 and A549, cells were selected using G418 (1 mg/ml) ∼72 h post transfection. All plasmids were generated in Dr J-C Bourdon’s laboratory (20).

### Assessing cell viability and IC_50_

Six hundred cells/well for H460, 700 cells/well for A549, and 1000 cells/well for all other cell lines were plated in 384-well plates (GreinerBio-one) in triplicates on the same plate. Cells were treated with DMSO or seven different concentrations of serially 3-fold-diluted NMDi, highest concentration being 9.6 μM, in a final volume of 40 μl. Seventy-two hours later, 11 μl of CellTiter Glo (CellTiter-Glo Luminescent Cell Viability Assay, REF G7573, Promega) was added, plates were shaken for 10 min, and luminescence was measured using a FLUOstar OPTIMA microplate reader (BMG LABTECH). Luminescence values were normalized to DMSO-treated cells. Each experiment was repeated two separate times to give biological replicates. IC_50_ values were estimated using drexplorer software, which fitted multiple dose–response models and identified the best model using residual standard error (40). In brief, the dose–response data were normalized by the mean response of controls prior to drug parameter estimation. Outlier data points were detected and removed as described in (40). The best dose–response model was selected based on residual standard error. Drug parameters including inhibitory concentrations (IC_10_∼IC_90_, original dose scale) were estimated using the drexplorer package (40). A paired *t* test was used to compare relative viability between NMDi-treated and DMSO-treated control groups.

For assessing cell viability and IC_50_ in p53-depleted A549 cells, cells transfected with either siControl or sip53 were used and treated as described above.

### Clonogenic survival assay

Exponentially growing cells were plated in duplicates at three dilutions into six-well plates containing 2 ml medium and incubated for 24 h in a humidified CO_2_ incubator at 37 °C. Subsequently NMDi was added into the medium for 16 h. After being pretreated with DMSO (for control) and NMDi, cells were subjected to 0, 2, 4, or 6 Gy of gamma irradiation by using a Mark 1-68A cell irradiator with a Cesium 137 source at a dose rate of 3.254 Gy per minute (J.L. Shepherd & Associates). The medium was then replaced with fresh medium allowing cells to continuously grow for colony formation for 10 to 14 days. To assess clonogenic survival following radiation exposure, cells were fixed with glutaraldehyde (6.0% v/v) and stained with crystal violet (0.5% w/v). Colonies with more than 50 cells were counted to determine percent survival, and the number of colonies obtained from three replicates was averaged for each treatment. These mean values were corrected according to plating efficiency of respective controls to calculate cell survival for each dose level. After correcting for plating efficiency, the survival fraction was calculated as reported (41) and fitted to a linear-quadratic model by using SigmaPlot 10.0. Clonogenic survival assays after irradiation were repeated two times for each cell line. For assessing radiation sensitivity of p53-depleted A549 cells, cells were transfected with either siControl or sip53; ∼24 h post transfection, cells were trypsinized and plated in triplicate (500 cells/well) in six-well plates; ∼ 48 h post transfection, cells were treated with either DMSO or NMDi (1 μM) for 16 h and subjected to radiation as described above. The experiment was repeated two times.

### Assessing colony-forming ability

For assessing colony-forming ability after UPF1 depletion, cells transfected with either siControl or siUPF1 were plated in duplicate at 200 cells per well concentration, in a six-well plate, approximately 26 h after transfection. For assessing colony-forming ability of NMDi-treated cells, cells were plated in triplicate at 100 cells per well concentration in a six-well plate and 24 h later treated with either DMSO or NMDi. For assessing colony-forming ability of p53-depleted A549 cells, cells transfected with either siControl or sip53 were plated in triplicate at 500 cells per well concentration, in a six-well plate, approximately 26 h after transfection; ∼24 h later, cells were treated with either DMSO or 1 μM NMDi. The medium was changed 24 h post treatment. About 10 to 15 days later, colonies were stained and counted as described above. The number of colonies obtained from three replicates was averaged for each treatment.

To assess the colony-forming ability of p53β- and p53γ-overexpressing H1299 cells, 100 cells per well were plated in duplicate in six-well plates and 10 days later, colonies were stained and the percent area covered by the colonies was quantitated using ImageJ (42). The experiment was repeated two separate times.

### RNA-Seq and mutation analysis

RNA-Seq and mutation data were analyzed as described (43). RNA expression for MDM2 and TP53 across all available cell lines in MD Anderson Cancer Center’s Lung database were integrated. The heat map was sorted by RNA expression of MDM2.

PanCancer tumor analysis for MDM2 amplification and TP53 mutation frequency were performed using the data from The Cancer Genome Atlas (TCGA) (44, 45) portal.

### Apoptosis assay

A total of 150,000 cells/well were plated in six-well plates. Approximately 24 h later, cells were treated with either DMSO or 3 μM NMDi for approximately 72 h. Cells were then trypsinized, washed two times with ice-cold phosphate-buffered saline (PBS), and processed for staining using FITC Annexin V Apoptosis Detection Kit with 7AAD (Cat # 640922, BioLegend), according to manufacturer’s instructions. Cells were analyzed by Flow Cytometry.

For assessing apoptosis in p53-depleted A549 cells, cells transfected with either siControl or sip53 (150,000 cells/well) were plated in triplicate in six-well plates and ∼ 24 h later treated with either DMSO or NMDi and processed as above. Experiments were repeated twice.

### Cell cycle analysis

One million cells were plated in 100-mm plates. Twenty-four hours later, cells were treated with either DMSO or NMDi (1 μM). Cells were harvested 24 and 48 h post treatment, fixed in 70% ethanol for 24 h at 4 °C, washed with 1× PBS, treated with 200 μg/ml RNase A in 1× PBS for 1 h at 37 °C, stained with 40 μg/ml propidium iodide for 20 min at room temperature, spun at 500*g* for 5 min to remove propidium iodide, and the cell pellet was resuspended in 1X PBS (500 μl). Fluorescence-activated cell sorting analysis was performed in BDFACSCanto flow cytometer. Data were analyzed using FlowJo-V10 (FlowJo, LLC, https://www.flowjo.com).

### Tumor growth assessment

All animal studies were approved by the Institutional Animal Care and Use Committee at the University of Texas MD Anderson Cancer Center in accordance with National Institutes of Health guidelines. Female mice (Strain 69 Athymic Nude (nu/nu) mice) were purchased from Envigo. Mice were randomly assigned to control or treatment groups. No statistical method was applied to predetermine the sample size, and the investigators performing preclinical experiments were not blinded. A549 (5 × 10^6^ cells per mouse) were injected subcutaneously into nude female mice. Mice were randomized for treatment (at least ten per group) when the tumor size reached ∼250 to 300 mm^3^. Mice were then treated with NMDi or vehicle for 3 weeks. NMDi (75 mg/kg) was administered on a 5-days-on and 2-days-off regimen. Tumors were harvested at the end of the treatment regimen and processed for RNA collection and RT-qPCR analysis.

### Statistical analysis

For assessing cell viability, a paired *t* test was used to compare relative viability between NMDi-treated and DMSO-treated control groups. For mRNA expression fold change analysis, unpaired *t* test (two-tailed) were used to compare the relative expression between NMDi-treated and DMSO-treated control groups. For analysis of tumor growth data, quantitative data were subjected to two-way analysis of variance (ANOVA).

## Data availability

All of the data are contained within the article.

## Supporting information

This article contains supporting information (43, 44, 45).

## Conflict of interest

T. C. has advisory role in MedImmune and research funding from Bristol-Myers Squibb and Boehringer Ingelheim, MedImmune. P. J. is the advisor of Tvardi Therapeutics and holds stock options in Tvardi. E. P. S. is on the advisory board and has research funding and travel support from Novocure; is on the advisory board and has travel support from BrainLab; is on the advisory board and has research funding from AbbVie; is on the advisory board of Blue Earth Diagnostics; and has speaker honoraria and travel support from Merck and speaker honoraria from PER. F. M. J. has received research funding from PIQUR Therapeutics and Trovagene. J. V. H. is the consultant/advisory board member for Bristol-Myers Squibb, AstraZeneca, Merck, Genentech, EMD Serono, Boehringer Ingelheim, Spectrum, Lilly, Novartis, and GSK. No potential conflicts of interest were disclosed by the other authors.
