# Supplementary material for: Inhibition of nonsense-mediated decay rescues p53β/γ isoform expression and activates the p53 pathway in MDM2-overexpressing and select p53-mutant cancers
Source: J Biol Chem. 2021 Sep 3;297(5):101163. doi: 10.1016/j.jbc.2021.101163 (PMC8569473; doi:10.1016/j.jbc.2021.101163)

**Inhibition of nonsense-mediated decay rescues p53β/γ isoform expression and activates the p53 pathway in MDM2-overexpressing and select p53-mutant cancers**

Jayanthi P. Gudikote^1^, Tina Cascone^1^, Alissa Poteete^1^, Piyada Sitthideatphaiboon^2^, Qiuyu Wu^3,^ Naoto Morikawa^4^, Fahao Zhang^1^, Shaohua Peng^1^, Pan Tong^5^, Lerong Li^5,6^, Li Shen^5^, Monique Nilsson^1^, Phillip Jones^7^, Erik P. Sulman^8^, Jing Wang^5,9^, Jean-Christophe Bourdon^10^, Faye M. Johnson^1,9^, and John V. Heymach^1,^*

**Supplementary information**

**Supplementary Figures**

**
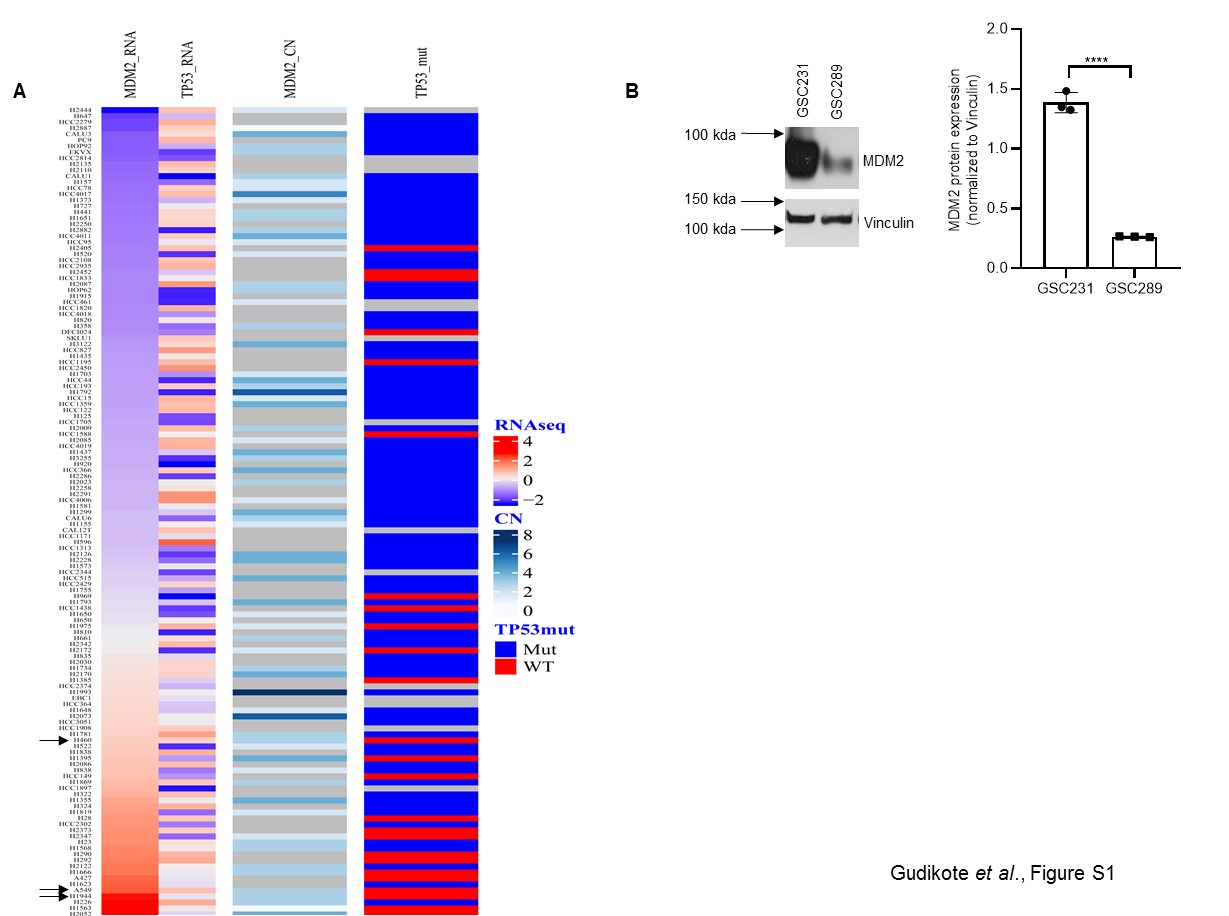
**

**Figure. S1. MDM2 copy number and expression status in NSCLC and GBM cell lines. (A)** mRNA expression and MDM2 copy number analysis of NSCLC cell lines. **(B)** Western blot showing MDM2 expression in GBM cell lines shown (left panel) and quantification of MDM2 protein expression (right panel). mRNA expression analysis done using RNAseq data, as described in Experimental procedures (43). *TP53* mutation status are indicated (Mut, mutant, WT, wild-type). CN, copy number. Arrows indicate the cell lines used in this study.

**
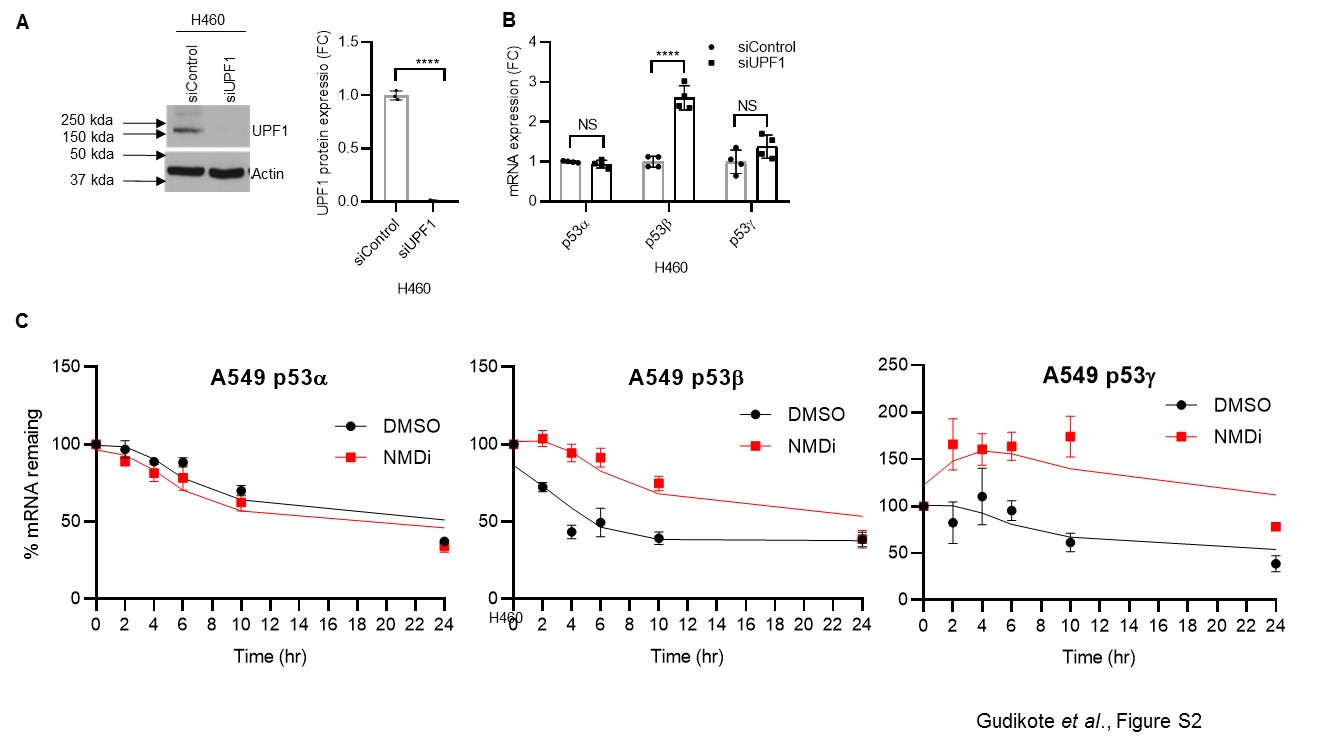
**

**Figure S2. NMD inhibition induces p53β/γ expression and prolongs p53β and p53γ mRNA decay.**

**(A)** Western blot showing UPF1 knockdown efficiency in H460 cells (left panel) and quantification of the blot (right panel).  **(B)** mRNA expression analysis of p53α, p53β, and p53γ in UPF1 depleted H460 cells. RT-qPCR analysis shown are average of 2 independent experiments with two technical repeats for each (n=4). Mean ± s.d., p values, two tailed t-tests, ****< 0.0001, NS, not significant. **(C)** mRNA decay analysis of p53α, p53β and p53γ transcripts from either DMSO or NMDi (1µM) treated A549 cells. RNA was extracted at the indicated time points after 5,6-Dichloro-1-β-D-ribofuranosylbenzimidazole (100µM) treatment as described in Experimental procedures. RT-qPCR analysis shown are mean ± s.d of three independent experiments.

**
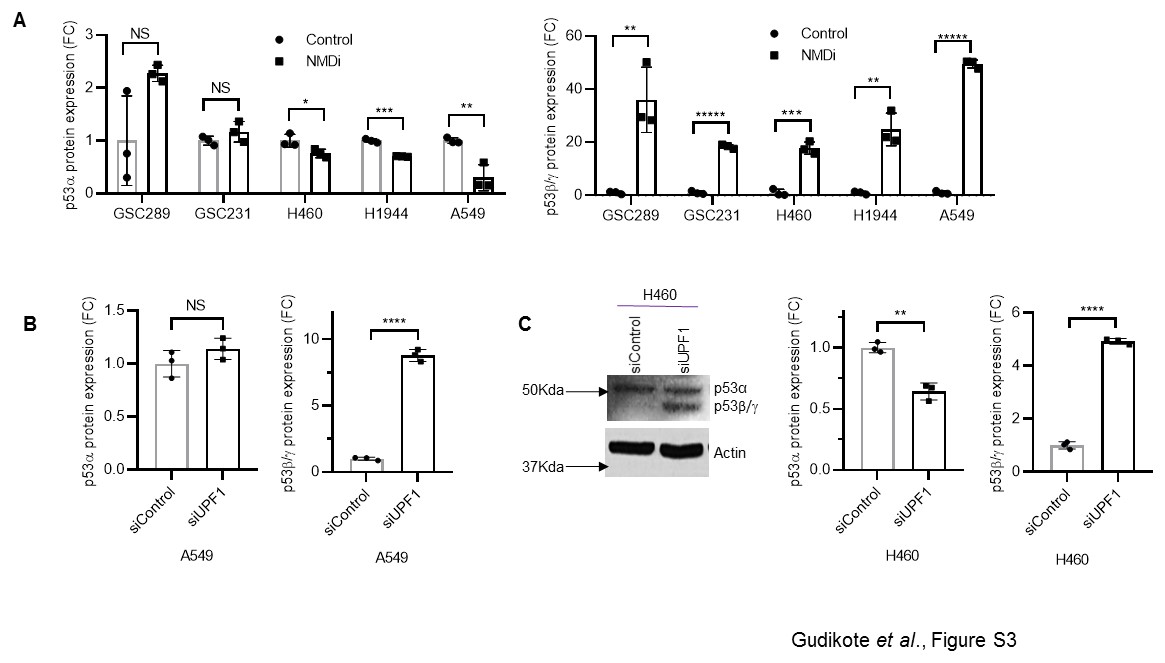
**

**Figure S3. Pharmacological or genetic inhibition of NMD induces p53β/γ protein expression. (A)** Quantification of p53α (left panel) and p53β/γ (right panel) protein expression in NMDi treated cell lines shown. **(B)** Quantification of p53α (left panel) and p53β/γ (right panel) protein expression in A549 cells treated with the indicated siRNAs.  **(C)** Western blot analysis (left panel) and quantification of p53α and p53β/γ protein expression (right panels) in H460 cells treated with the indicated siRNAs. H460 Western blot from figure S2A was reprobed with p53 antibody to evaluate the expression of p53 isoforms. Protein quantifications shown (n = 3) are from Western blots from three independent experiments. Mean ± s.d., p values, two tailed t-tests, *≤0.05, **< 0.01, ***< 0.001, ****< 0.0001, *****< 0.00001, NS, not significant.

**
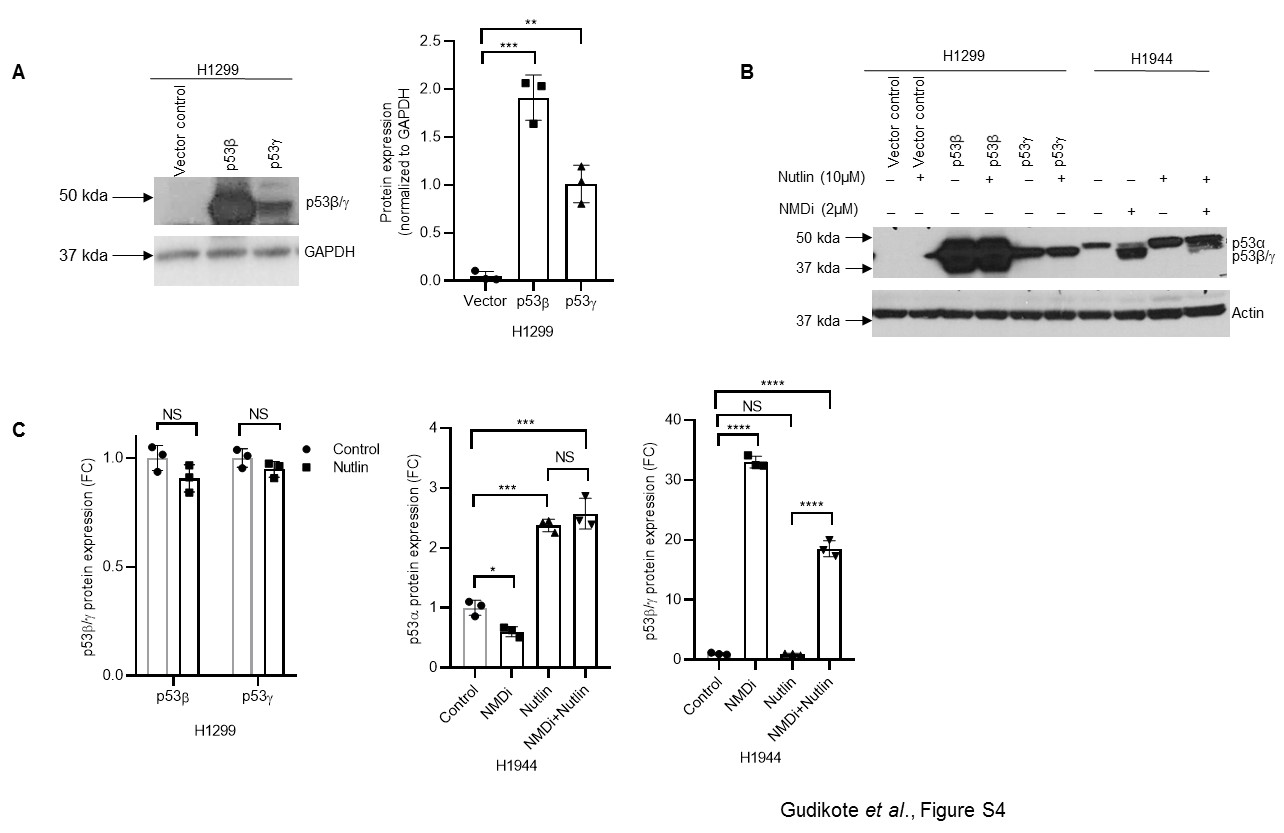
**

**Figure S4. MDM2 inhibition increases p53α but not p53β/γ expression.**

**(A)** Western analysis showing overexpression of p53β and p53γ isoforms (left panel) and quantification of p53β and p53γ proteins (right panel) in H1299 cells. **(B)** Western analysis of H1299 overexpressing p53β/p53γ and H1944 treated with the indicated drugs. H1944 cells were treated either with DMSO or with NMDi or with nutlin or with a combination of NMDi and nutlin as descibed in the Experimental procedures. Data from lanes 7 and 8 (H1944, –NMDi and +NMDi) showing the expression of p53β/γ in H1944 upon NMDi treatment is shown in Figure 1F. In current figure, these lanes are reused to compare the expression of p53β/γ in the presence and absence of nutlin. **(C)** Quantification of protein bands shown in B. Protein quantifications shown (n = 3) are from Western blots from three independent experiments. Mean ± s.d., p values, two tailed t-tests, *≤0.05, **< 0.01, ***< 0.001, ****< 0.0001, NS, not significant.

**
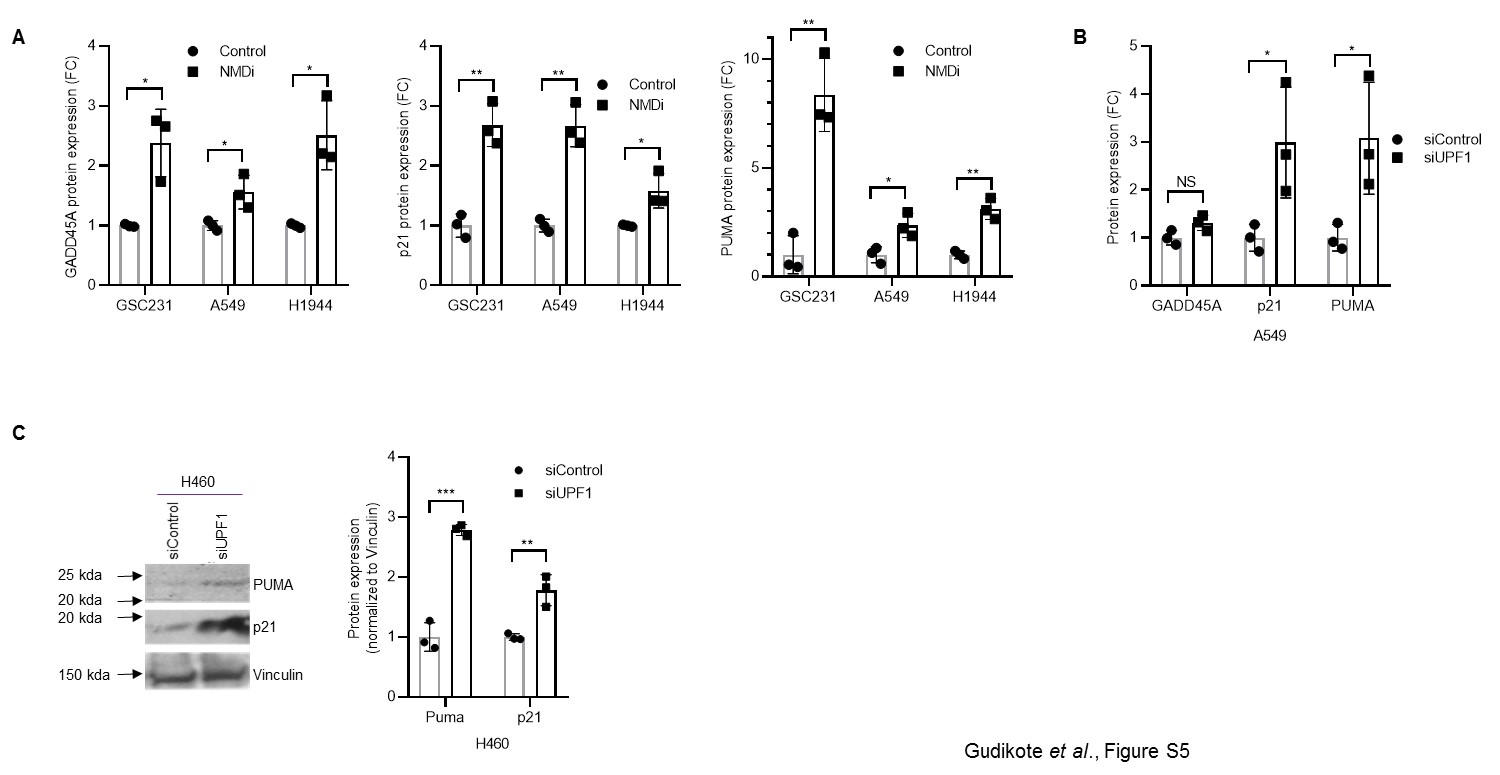
**

**Figure S5. NMD inhibition upregulates the expression of p53 transcriptional targets. (A)** Quantification of p53 transcriptional target protein expression in indicated cell lines treated with NMDi. **(B)** Quantification of p53 transcriptional target protein expression in A549 cells treated with the indicated siRNAs. **(C)** Western analysis (left panel) and protein quantification (right panel) of p53 transcriptional targets in UPF1-depleted H460 cells. Mean ± s.d., p values, two tailed t-tests, *≤0.05, **< 0.01, ***< 0.001, NS, not significant.

**
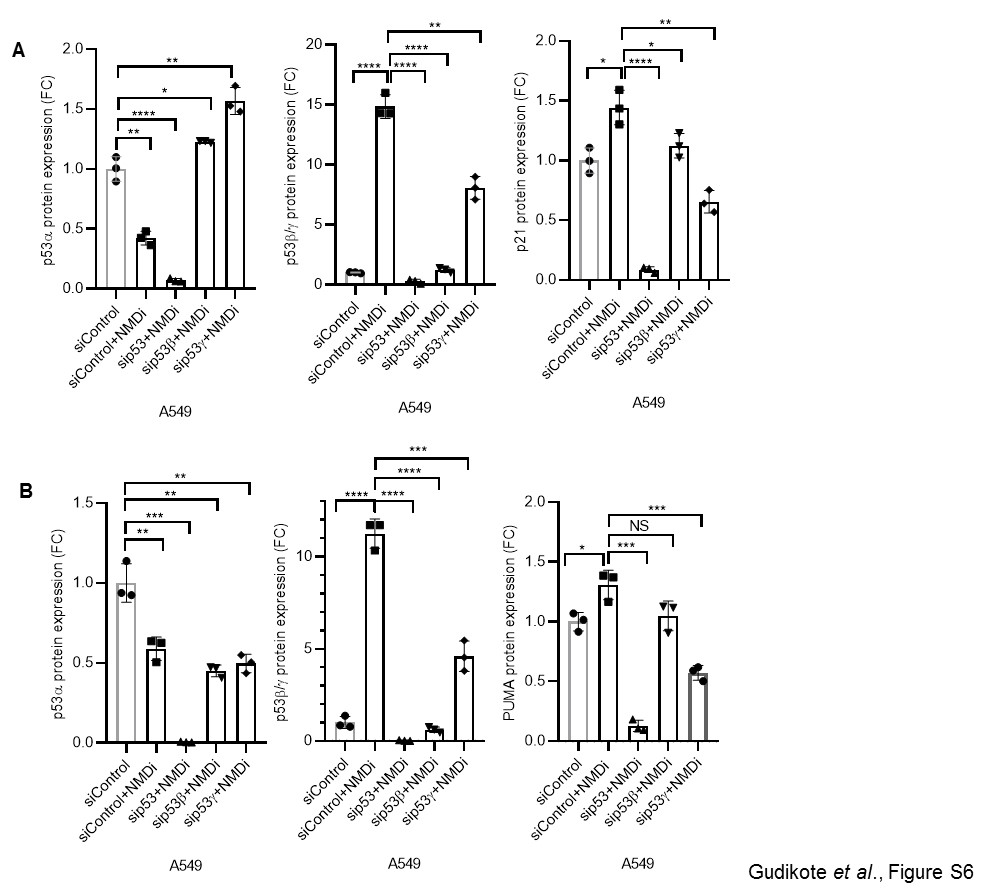
**

**Figure S6. NMD inhibition-induced upregulation of p21 and PUMA is p53-dependent. (A)** Quantification of p53α (left panel), p53β/γ (middle panel) and p21 (right panel) protein expression in A549 cells treated with the indicated siRNAs and NMDi. **(B)** Quantification of p53α (left panel), p53β/γ (middle panel) and PUMA (right panel) protein expression in A549 cells treated with the indicated siRNAs and NMDi. Protein quantifications shown (n = 3) are from Western blots from three independent experiments. Mean ± s.d., p values, two tailed t-tests, *≤0.05, **< 0.01, ***< 0.001, ****< 0.0001, NS, not significant.

**
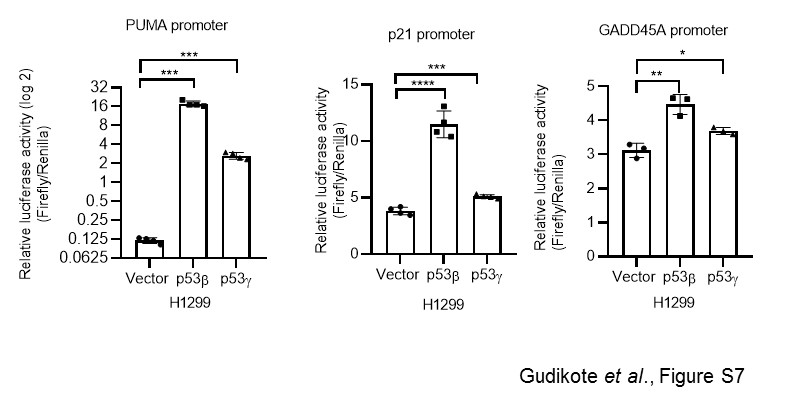
**

**Figure S7. p53β and p53γ overexpressing H1299 cells show increased binding to promoters of p53 transcriptional targets.** H1299 cells were transfected with the indicated cDNAs and with luciferase reporter constructs containing either PUMA or p21 or GADD45A promoter sequences. Relative luciferase activity is mean ± s.d of at least 3 independent experiments. Mean ± s.d., p values, two tailed t-tests, *≤0.05, **< 0.01, ***< 0.001, ****< 0.0001.

**
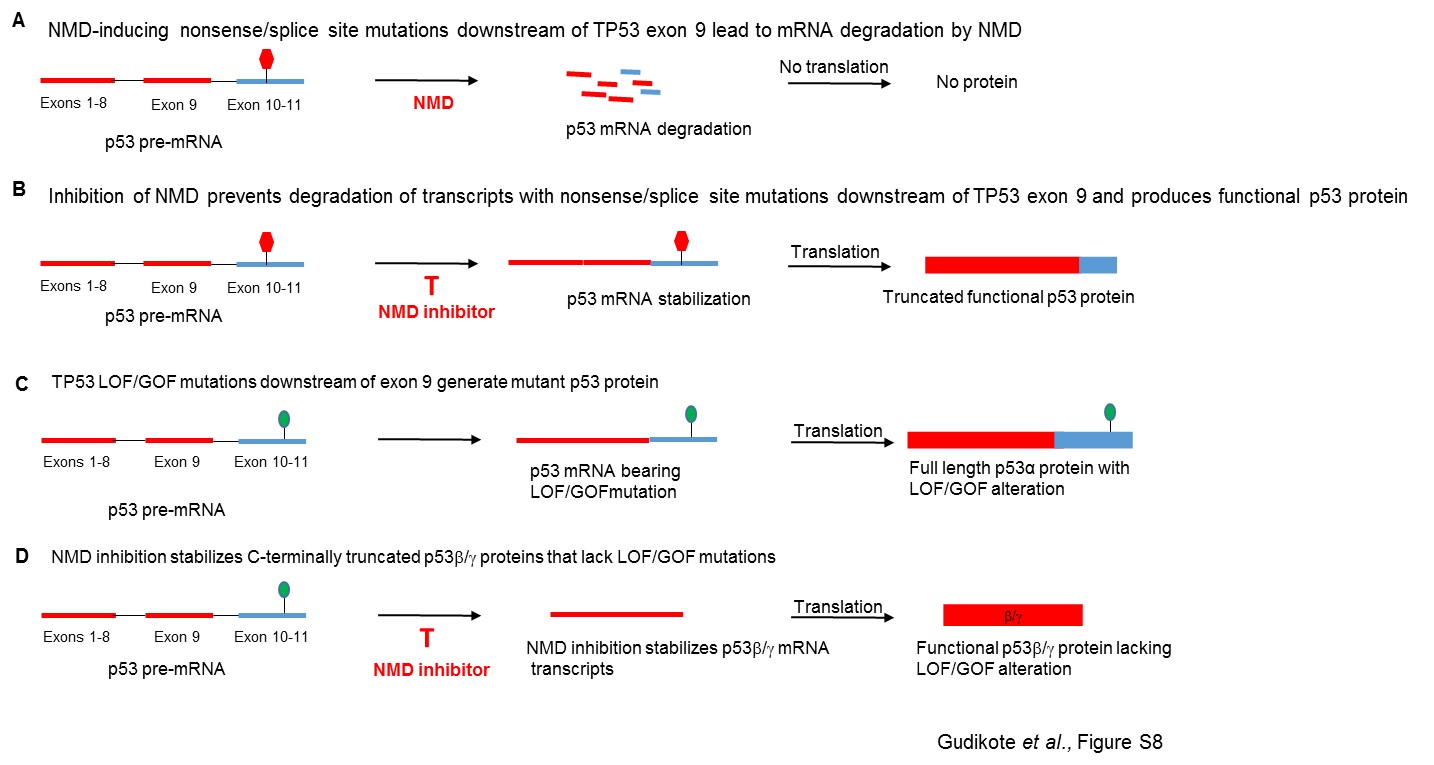
Figure S8. p53 function restoration strategy in transcripts bearing mutations downstream of exon 9.**

**(A)** In the absence of NMD inhibition, NMD-inducing nonsense or splice site mutations downstream of p53 exon 9 elicit mRNA degradation. **(B)** By inhibiting NMD, p53 transcripts bearing NMD-inducible mutations downstream of exon 9 can be protected and translated into near full-length functional proteins. **(C)** Missense mutations downstream of exon 9 can generate mutant p53 protein with LOF/GOF alteration. **(D)** NMD inhibition can potentially overcome the effect of LOF/GOF mutations downstream of exon 9 by stabilizing the expression of functional p53β/γ which are C-terminally truncated and hence, lack the mutation. Mutations generating PTCs are depicted as red stop signs and missense mutations which attribute LOF/GOF are depicted in green.

**
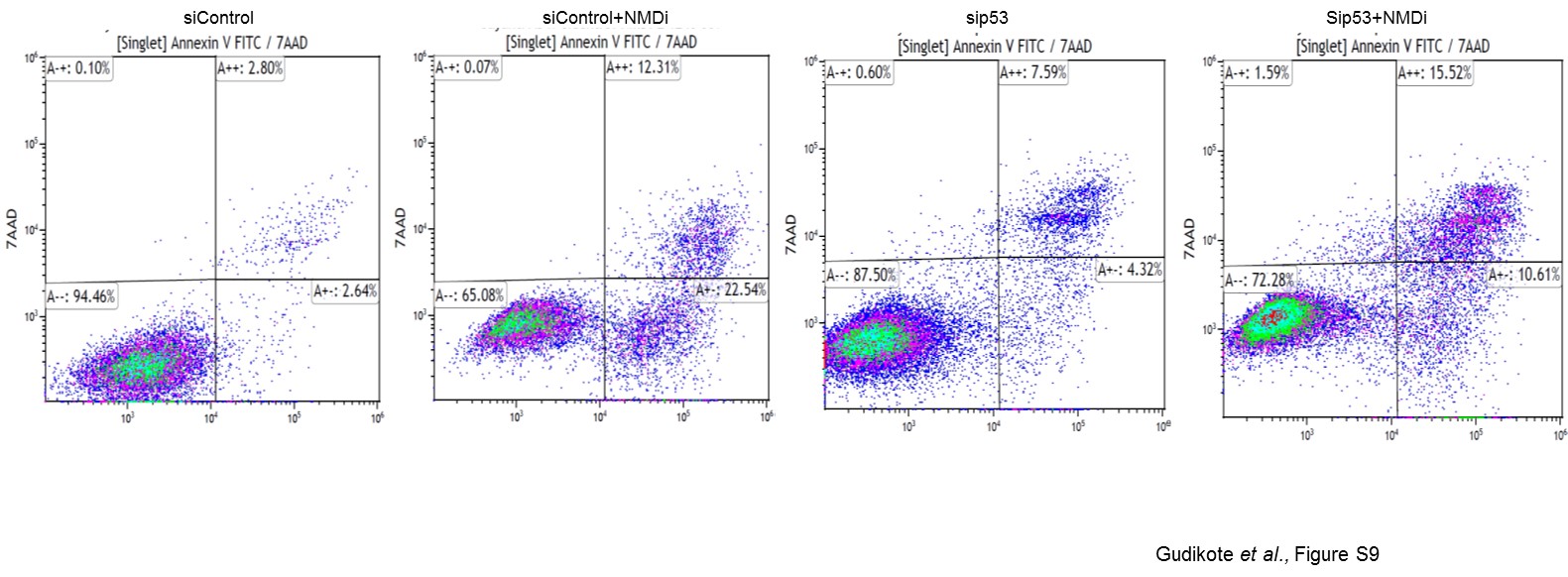
**

**Figure S9. Depletion of p53 mitigates NMDi-induced apoptosis**. FACS analysis of A549 cells treated with either siControl or sip53 and, either DMSO or NMDi. Numbers in quadrant A^--^ show percent of live cells, quadrant A^+^ indicate percent of cells in early apoptotic phase and quadrant A^++^ indicate percent of cells that have undergone apoptosis. Quadrant A^-+^ indicate percent of necrotic cells. Data shown are representatives of two independent experiments.

**
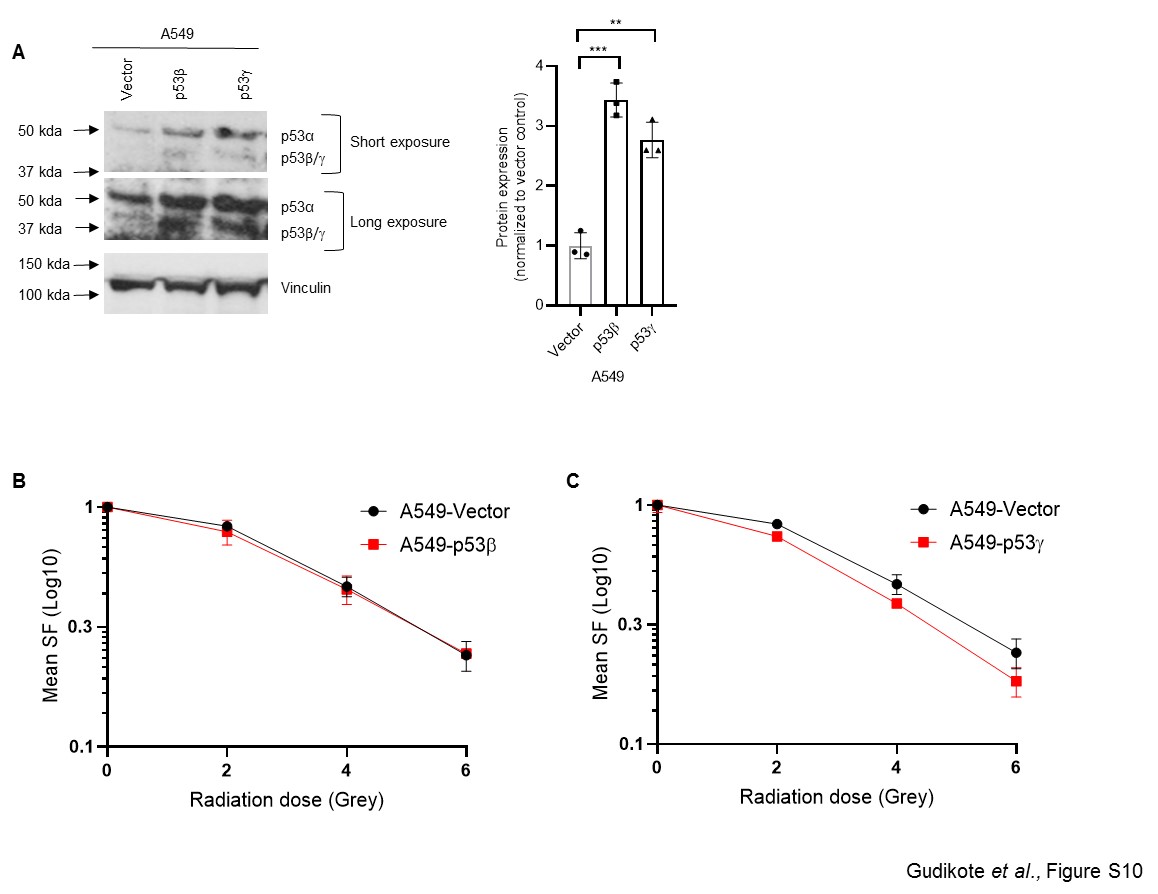
**

**Figure S10. p53γ overexpression shows modest increase in radiation sensitivity in A549 cells.**

**(A)** Western blot showing p53β and p53γ overexpression in A549 stably transfected with either vector control or p53β or p53γ cDNA constructs (left panel) and quantification of the Western blot (right panel). Mean ± s.d., p values, two tailed t-tests, **< 0.01, ***< 0.001. **(B)** and **(C)** Radiation sensitivity of A549 cells overexpressing vector control or p53β **(B)** and vector control or p53γ **(C)**. Data shown is the mean±s.e., n=3. SF, Survival fraction.

**
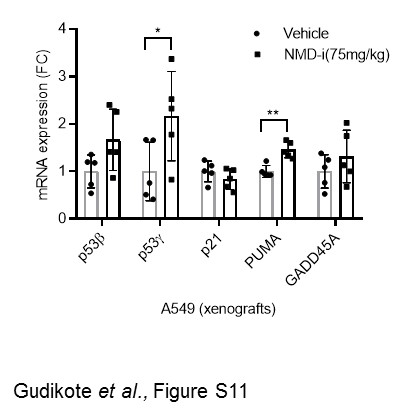
**

**Figure S11. NMDi increases the expression of p53γ and p53 transcriptional targets in A549 xenograft tumors.**

mRNA expression FC of the indicated transcripts in vehicle or NMDi treated tumor tissue samples derived from A549 subcutaneous xenografts. Tumors were harvested at the end of the treatment, mRNA expression assessed by RT-qPCR. Mean ± s.e., n = 5 each, p values, two tailed t-tests, *≤0.05, **< 0.01.

**Supplementary Tables**


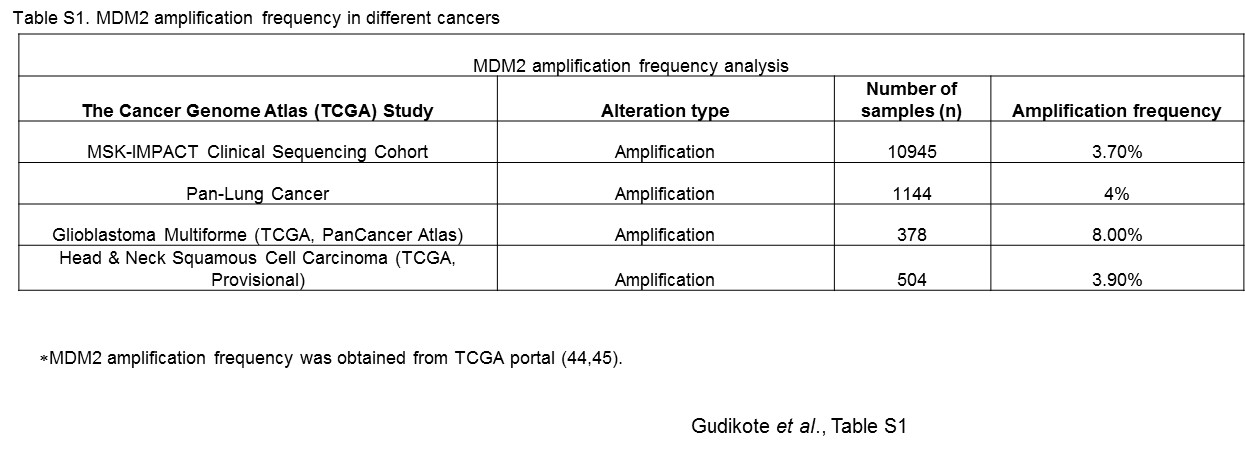


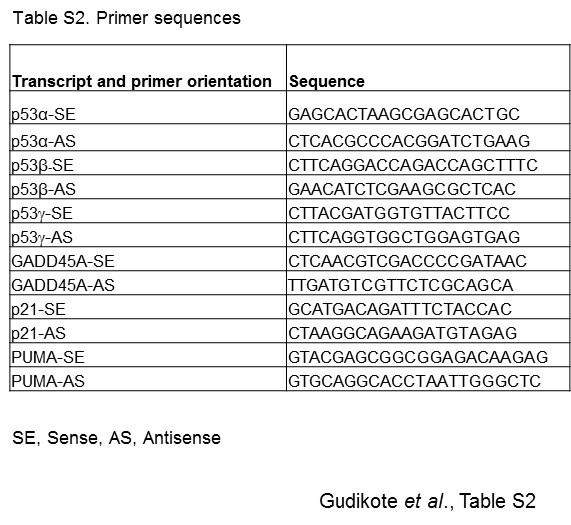


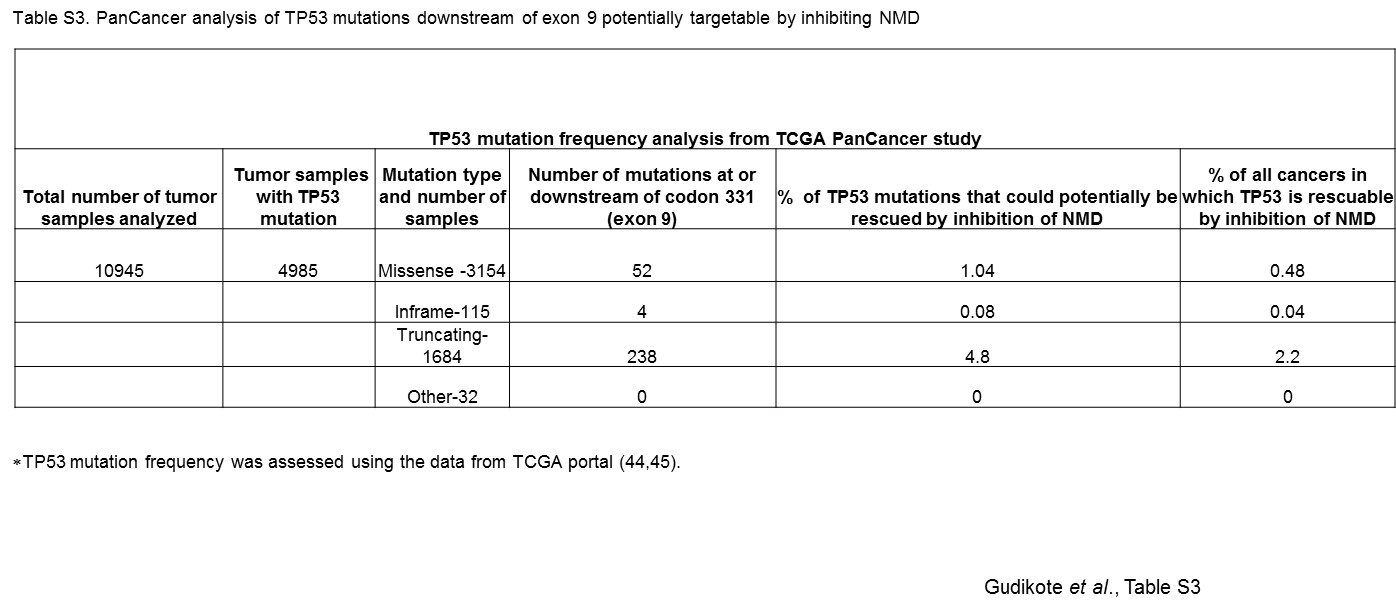

Supplement: Supplemental Figures S1–S11 and Tables S1–S3 [file mmc1.docx]
